# Theory of isolated magnetic skyrmions: From fundamentals to room temperature applications

**DOI:** 10.1038/s41598-018-22242-8

**Published:** 2018-03-13

**Authors:** Felix Büttner, Ivan Lemesh, Geoffrey S. D. Beach

**Affiliations:** Department of Materials Science and Engineering, Massachusetts Institute of Technology, Cambridge, Massachusetts, 02139 USA

## Abstract

Magnetic skyrmions are topological quasiparticles of great interest for data storage applications because of their small size, high stability, and ease of manipulation via electric current. However, although models exist for some limiting cases, there is no universal theory capable of accurately describing the structure and energetics of all skyrmions. The main barrier is the complexity of non-local stray field interactions, which are usually included through crude approximations. Here we present an accurate analytical framework to treat isolated skyrmions in any material, assuming only a circularly-symmetric 360° domain wall profile and a homogeneous magnetization profile in the out-of-plane direction. We establish the first rigorous criteria to distinguish stray field from DMI skyrmions, resolving a major dispute in the community. We discover new phases, such as bi-stability, a phenomenon unknown in magnetism so far. We predict materials for sub-10 nm zero field room temperature stable skyrmions suitable for applications. Finally, we derive analytical equations to describe current-driven dynamics, find a topological damping, and show how to engineer materials in which compact skyrmions can be driven at velocities >1000 m/s.

## Introduction

Magnetic skyrmions are spin configurations with spherical topology^1–5^, typically manifesting as circular domains with defect-free domain walls (DWs) in systems with otherwise uniform out-of-plane magnetization. Skyrmions are the smallest non-trivial structures in magnetism and they behave like particles^6–11^, which makes them of fundamental interest and of practical utility for high-density data storage applications^12–15^. Skyrmions have been investigated for decades^16^, but only recently has attention shifted to the detailed domain wall structure and thereby-determined topology. Two factors have driven this trend: technological advances allowing for direct imaging of the spin structure^17–19^ and the discovery that the Dzyaloshinskii-Moriya interaction (DMI) can be used to stabilize that structure. In particular, DMI can lead to a skyrmion global ground state above the Curie temperature (*T*_*c*_) in a Ginzburg-Landau theory of a ferromagnet^20,21^. Stray fields are not included in that model, but are of critical importance for understanding skyrmions in real materials, as underlined by the fact that all isolated room temperature skyrmions so far were observed in relatively thick films with sizable saturation magnetization^11,22–28^ and hence very strong stray field interactions. To explore the full skyrmion phase diagram and understand skyrmion stability, a theory with accurate predictive power is required and such a theory must include stray field interactions.

Stray field energies are the most difficult to treat analytically due to their nonlocal nature: they involve six-dimensional integrals whose kernel locally diverges. All existing models involve approximations that are motivated only by the need to simplify, without considering the error of the approximation or the limits of validity^16,20,29–37^. Tu^38^, Kiselev *et al*.^39^, and Guslienko^31^ evaluated the full stray field integrals, but only numerically, which is prohibitively slow and technically demanding. Micromagnetic simulations are hence the only generally applicable tool to obtain quantitative predictions, but they are too slow to systematically examine skyrmion properties across a parameter space comprising four material parameters, film thickness, and magnetic field.

Here, we derive a fully analytical theory with the precision of micromagnetic simulations but orders of magnitude faster performance, providing unique access to the full skyrmion phase diagram and deep insights into the underlying physics. For instance, we can mathematically prove that there are two types of skyrmions, stray field skyrmions and DMI skyrmions, and we discuss how to experimentally distinguish between them. Our theory predicts a sharp transition separating these two skyrmion phases, and a phase pocket in which they can co-exist, leading to bi-stabilities and zero-stiffness skyrmions. These new states exist at room temperature and can have many novel applications, some of which we suggest here. In view of applications, we prove that the Co-based multilayers at the focus of most current experimental efforts are incapable of hosting room-temperature-stable sub-10nm skyrmions. However, by examining >10^6^ material parameter combinations, we identify alternative materials suitable to host such skyrmions at room temperature without a stabilizing field. Finally, we derive simple analytical equations to describe current-driven skyrmion dynamics with accuracy comparable to micromagnetics but yielding key insights that cannot be gained from numerics. We discover a topological contribution to damping that severely reduces the mobility of small skyrmions, and propose materials that can mitigate this effect to permit sub-10 nm skyrmions to be driven by current at >1 km/s with vanishing skyrmion Hall angle.

## Results

### Validation of the model

Our theory predicts the energy of isolated skyrmions in a film of arbitrary thickness and infinite in-plane extent, based on the recent experimental confirmation^19,23^ of an analytic and universal 360° domain wall (DW) model^40^ for the spin structure of all skyrmions. Although the 360° DW model is not an exact solution of the micromagnetic energy functional, it shows excellent agreement with experiments^19,23^ and is validated by our extensive micromagnetic simulations. Taking this model as an ansatz for the skyrmion spin structure, we derived analytic expressions for the total energy function with better than 1% accuracy over the entire parameter space (see Supplementary Information), assuming only that the structure does not vary along the out-of-plane direction. For a given set of material parameters (uniaxial anisotropy constant *K*_*u*_, saturation magnetization *M*_*s*_, exchange constant *A*, interface and bulk DMI strengths *D*_*i*_ and *D*_*b*_, and magnetic layer thickness *d*) and external field *H*_*z*_, minimization of this function yields the equilibrium skyrmion configuration. Multilayers are included straightforwardly via the effective medium approach^24,41^ (for the limitations of the effective medium model, see ref.^41^). Our model is therefore generally applicable to skyrmions in any material, with the exception of thick multilayers with very strong stray fields and weak DMI where flux closure surface states form and where the assumption of uniform magnetization along the out-of-plane direction does not hold^42^. In the Supplementary Information, we provide analytical expressions for both bulk and interface DMI terms; in the discussions below, we apply our model with a focus on systems with interface DMI only, but the model itself is more general.

**Figure 1:**
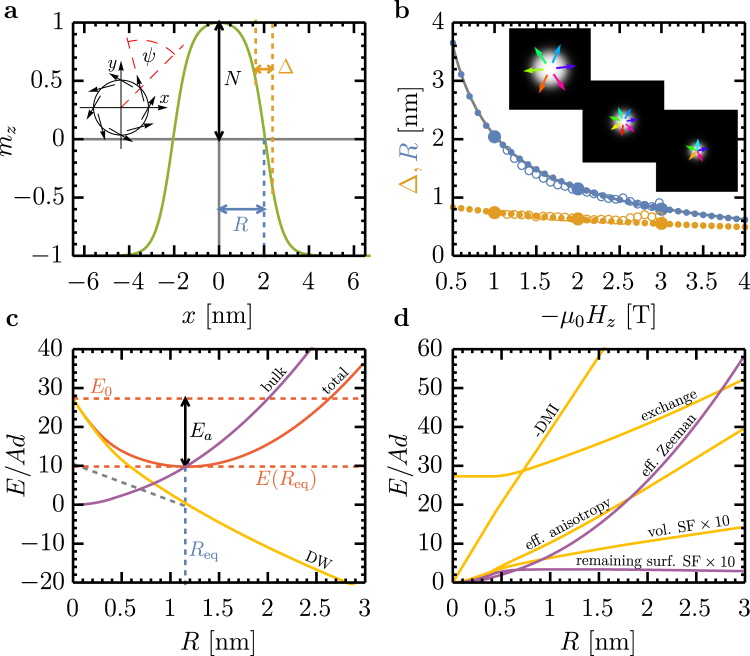
Skyrmion profile and stabilizing energy. (**a**) Illustration of the 360° domain wall model. The plot shows the normalized perpendicular magnetization *m*_*z*_ as a function of position *x* along the diameter of a skyrmion and defines the characteristic parameters *R* (radius), Δ (domain wall width), *ψ* (domain wall angle, inset), and *N* (polarity or skyrmion charge). The profile corresponds to the *μ*_0_*H*_*z*_  = −1 T data point in (**b**). The negative value of the field indicates that it is oriented antiparallel to the skyrmion core. (**b**) Radius and domain wall width as a function of applied magnetic field *μ*_0_*H*_*z*_. The small solid data points are predictions of our analytical model, the solid large data points are results from micromagnetic simulations, the open points are experimental results of Romming *et al*.^19^. The insets show the relaxed spin structures obtained from micromagnetic simulations corresponding to the large solid data points. (**c**) Total energy, domain wall energy, and bulk energy as a function of skyrmion radius at a field of *μ*_0_*H*_*z*_ = −2 T. At each value of *R* the energy has been minimized to determine Δ and *ψ*. *R*_eq_ is the equilibrium radius as plotted in (**b**). At the minimum, the domain wall energy has a negative slope, qualifying this skyrmion as a DMI skyrmion. (**d**) Decomposition of the total energy in **c** into individual components. Domain wall energies (yellow): DMI energy (inverted), exchange energy, effective anisotropy energy, and volume stray field energy (multiplied by 10). Bulk energies (pink): effective Zeeman energy and remaining surface stray field energies (multiplied by 10).

The spin structure is parameterized by its radius *R*, domain wall width Δ, domain wall angle *ψ*, and topological charge *N* (Fig. 1a). *R* and Δ determine the magnetization profile *m*_*z*_(*x*, *y*), whereas *ψ* specifies whether the in-plane component of the domain wall spins is radial (Néel, *ψ* = 0, *π*), azimuthal (Bloch, *ψ* = *π*/2, 3*π*/2), or intermediate. Although the original 360° domain wall model included an analytical function Δ(*R*)^40^, we find that stray field interactions lead to a highly-nontrivial dependence of Δ on *R* and they must be treated independently. For large *ρ* = *R*/Δ, skyrmions consist of an extended out-of-plane magnetized domain bounded by a narrow circular domain wall, while for $\rho  \sim 1$ the inner domain is reduced to a point-like core. We refer to these limiting cases as bubble skyrmions and compact skyrmions^43^, respectively, consistent with the literature, but note that many skyrmions observed recently^11,22,24,44^ showed intermediate values of *ρ* and cannot be classified distinctly.

Figure 1b shows that our model agrees accurately with micromagnetic simulations and the experimental data of Romming *et al*.^19^. Our model yields the energy of a given skyrmion configuration in less than a millisecond on a regular personal computer and the full energy landscape in a few seconds, providing dramatic improvement over micromagnetic simulations. Moreover, it provides information not easily accessible by simulations. For example, since Δ and *ψ* can be minimized for any non-equilibrium *R*, one readily obtains the function *E*(*R*) that describes skyrmion stability and rigidity. By contrast, standard micromagnetic simulations only yield the equilibrium spin structure.

Figure 1c shows *E*(*R*) for the skyrmion in Fig. 1b at *μ*_0_*H*_*z*_ = −2 T, giving insight into skyrmion stability. The individual energy contributions are plotted in Fig. 1d. There are two stable states: the skyrmion state at *R*_eq_ and the ferromagnetic ground state at *R* = 0. Despite their different topology, there is a path from one to the other through the singular *R* = 0 state. This singular state lacks a topology, hence topological quantization is lifted here. As seen in Fig. 1d, all energy terms vanish as *R* → 0 except for the exchange energy, leading to a finite, universal skyrmion energy at *R* = 0,

1 $$E(R=0)={E}_{0}\approx 27.3Ad.$$

This value is close to the absolute minimum 8*πAd* of the exchange energy of a continuous spin structure with integer topological charge^3,36^, and much smaller than the 38*Ad* predicted using a simple linear skyrmion profile^33^. Our identification of a topologically valid and energetically possible path to annihilation clarifies that all skyrmions can be annihilated, even in continuum models, despite the common perception that topological protection guarantees stability^32,45,46^. In particular, although *E*_0_ relates to topology as previously discussed by Belavin and Polyakov^3^, it corresponds to the skyrmion nucleation energy barrier *E*_*n*_. Skyrmion stability, however, is related to the annihilation energy barrier *E*_*a*_ = *E*_0_ − *E*(*R*), which depends nontrivially on all micromagnetic parameters and has no direct topological origin.

The annihilation barrier in Fig. 1c corresponds to only $\sim 3{k}_{B}{T}_{{\rm{RT}}}$ at *T*_RT_ = 300 K, and hence the small skyrmions observed by Romming *et al*.^19^ can only exist at cryogenic temperatures. We focus in the remainder of this work on room-temperature stable skyrmions, i.e., those with *E*_*a*_ > 50*k*_*B*_*T*_RT_ corresponding to lifetimes >10 years, as required for applications. We note that *E*_*n*_ and *E*_*a*_ serve as upper bounds for the true energy barriers because skyrmions can deform in a way that is not covered by the 360° domain wall model, hence reducing the nominal energy barrier^47^. However, previous studies^47^ and our own micromagnetic simulations indicate that deformations reduce the energy barrier by <2*Ad*. Note that the collapse energy *E*_0_ in the atomistic simulations by Rohart *et al*.^47^ is 23*Ad* and 22*Ad* with and without deformations, respectively, significantly smaller than the continuum limit of 8*πAd*, which the authors attribute to lattice effects. Such effects are beyond the scope of the present continuum theory, and therefore, we limit our subsequent discussion to the validity range of the micromagnetic framework, i.e., to diameters of approximately 1 nm and larger, and subtract 2*Ad* from all energy barriers in an attempt to include possible deformations.

### Smallest room-temperature stable size – unifying existing models

**Figure 2:**
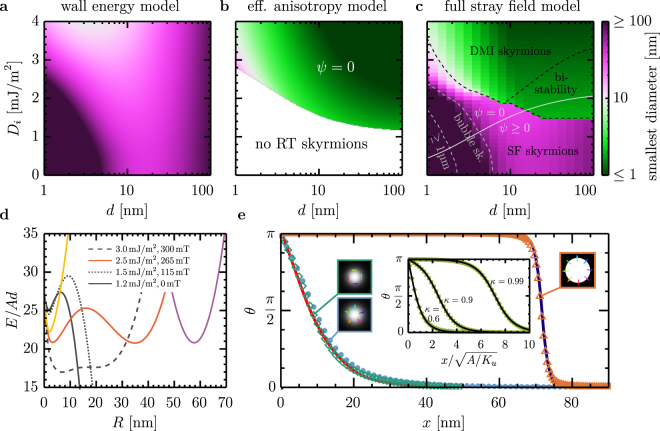
Fundamental predictions of our model. (**a–c**) Room temperature collapse diameter predicted by different models (always requiring 50*k*_*B*_*T* for stability, but all diagrams would look very similar for 30*k*_*B*_*T*). In all diagrams, *d* is the thickness of the magnetic material and the total film thickness (including non-magnetic spacer layers) is 4*d*. All three diagrams were derived with *A* = 10 pJ/m, *Q* = 1.4, *M*_*s*_ = 1.4 MA/m, and *H*_*z*_ such that *R* is minimum. (**a**) Wall energy model of bubble domains^48^. (**b**) Bogdanov and Hubert’s model^34,35^, which considers stray fields by effective anisotropies. (**c**) Our model including stray field in their full non-local nature. (**d**) Characteristic energy plots for different values of *D*_*i*_ at *d* = 31.6 nm. The yellow and pink solid lines are the domain wall and bulk energy corresponding to the red solid line, respectively. The bulk energy has been offset by 155*Ad* to present it in the given plot range. (**e**) Radial cross section of a simulated non-DMI skyrmion (green) and low DMI (stray field) skyrmion (light blue). The red and blue lines are reproduced from ref.^39^, where the attempt was made to distinguish proper skyrmions from stray-field stabilized bubbles based on their radial profile. The radial profile of the simulated stray field skyrmions precisely agrees with the suggested shape of a DMI skyrmion, demonstrating that radial profile is not a valid selection criterion. *θ* = arccos (*m*_*z*_) is the out-of-plane spin angle. The inset shows a fit of the 360° domain wall model to the numerically obtained solutions of the Euler equation of a zero-*M*_*s*_ system^34^. The following parameters were used for the simulations: *d* = 2.8 nm, *A* = 10 pJ/m, *M*_*s*_ = 1.4 MA/m, *Q* = 1.01, *D*_*i*_ = 0, *μ*_0_*H*_*z*_ = −63 mT for the non-DMI skyrmion and *d* = 1 nm, *A* = 10 pJ/m, *M*_*s*_ = 1.4 MA/m, *Q* = 1.01, *D*_*i*_ = 0.8 mJ/m^2^, and *μ*_0_*H*_*z*_ = −50 mT for the low DMI skyrmion (and no non-magnetic spacer layers in both cases).

Figure 2 shows that our model reproduces the limiting cases treated previously in the literature and continuously connects them to provide otherwise inaccessible insights into the entire skyrmion phase diagram. Two common approximations exist to describe circular spin textures in out-of-plane magnetized films. The first is the wall energy model^48^ introduced in the 1970s to treat bubble domains. This model approximates the domain walls as infinitely thin with constant energy per unit length *σ*_DW_, enabling exact solutions for the stray field energies^48^. A characteristic feature is that *E*(*R*) starts from zero with positive slope at *R* = 0 and goes through a maximum before reaching a minimum at *R* = *R*_eq_. This minimum vanishes above a critical field and the collapse diameter is finite even at *T* = 0. The second model, derived by Bogdanov and Hubert^34,35^ for small skyrmions in ultrathin films, approximates stray field energies as effective anisotropies. The original model does not include analytic solutions, but we derive these equations in the Supplementary Information. The effective anisotropy model predicts isolated skyrmions for any finite DMI^34^, and they cannot be annihilated by applied fields at *T* = 0.

The room temperature collapse diameter as a function of *D*_*i*_ and film thickness is shown in Figs 2a,b for the wall-energy model and effective anisotropy model, respectively, using the most accurate available form of the former^41^. These figures correspond to a 50*k*_*B*_*T*_RT_ stability criterion; a 30*k*_*B*_*T*_RT_ criterion (lifetime of order seconds) leads to similar results. The wall energy model predicts room-temperature stable skyrmions for all *D*_*i*_ and *d*, with a variable but size-independent *ψ*. These skyrmions are always relatively large. The effective anisotropy model, by contrast, predicts room-temperature stable skyrmions with size <10 nm, but only above a critical *D*_*i*_ that is quite large in ultrathin films. With interfacial DMI, skyrmions in this model are always purely Néel (see Supplementary Information).

The predictions for our theory are shown in Fig. 2c. The most obvious feature is a sharp boundary separating $\ll 10\,{\rm{nm}}$ and $\gg 10\,{\rm{n}}{\rm{m}}$ skyrmions. This phase boundary qualitatively follows the stability boundary in Fig. 2b, and the predicted collapse diameter above and below this contour agrees surprisingly well with the effective anisotropy and the wall energy model, respectively. It would therefore be tempting to use Bogdanov and Hubert’s model whenever it predicts room temperature stable skyrmions and the wall energy model in all other cases. This hybrid approximation would predict a collapse diameter diagram similar to the correct result in Fig. 2c, but there are important properties that this approach would omit. First, the transition from one model to the other is not always as sharp as the collapse-diameter figure suggests. At fields below the collapse field, large and small skyrmions can coexist, yielding the bi-stable region denoted in Fig. 2c. A hybrid model would be intrinsically incapable of describing this phase since it can only describe one type of skyrmion at a time. Second, the domain wall width and domain wall angle can strongly depend on the skyrmion radius, which is not predicted by either model.

Figure 2d shows how *E*(*R*) evolves with increasing DMI and how mixed states appear. Without DMI, *E*(*R*) starts with zero or positive slope at *R* = 0, then reaches a maximum and diverges logarithmically to −∞ if *H*_*z*_ = 0. An applied field can compensate the divergent stray fields and stabilize a minimum at large *R* (not shown in the figure). With DMI, the situation changes qualitatively: the slope at *R* = 0 becomes negative so the minimum appears before the maximum. Initially, the minimum is too shallow and appears in condition of unstable Δ (see Supplementary Information section 6). This is the case for *D*_*i*_ = 1.2 mJ/m^2^ in Fig. 2d. For larger *D*_*i*_, the minimum becomes deeper and shifts to larger *R*. At first, this new minimum is stable only at low fields (*D*_*i*_ = 1.5 mJ/m^2^ in Fig. 2d) but collapses before the stray field minimum at large *R* vanishes. That is, the smallest skyrmions are not necessarily found at the largest possible field values. At even larger *D*_*i*_, the minimum at small *R* becomes more pronounced and can become energetically degenerate with a second minimum, as shown for *D*_*i*_ = 2.5 mJ/m^2^ and *D*_*i*_ = 3.0 mJ/m^2^. The important difference between these two states is that the energy barrier separating the minima is much larger than 50*k*_*B*_*T*_RT_ for the lower DMI value (*D*_*i*_ = 2.5 mJ/m^2^) and almost vanishingly small for the larger *D*_*i*_. Those states constitute a bi-stability and a zero stiffness skyrmion, respectively, as discussed in more detail below. At even larger DMI, the maximum between the two minima vanishes and only one stable state remains.

The spin textures in Fig. 2c are all topologically equivalent: they exhibit spherical topology and unity topological charge and hence are all magnetic skyrmions. However, there has been much disagreement in the literature as to whether different stabilization mechanisms lead to fundamentally different types of skyrmions, and, if so, how to distinguish between them. Kiselev *et al*.^39,49^ suggested to use the skyrmion profile to determine its type. Similar to Guslienko’s study on stray field skyrmions in magnetic dots^31^, we find that the radial profile is not a robust distinguishing criterion, as shown in Fig. 2e. We find that stray fields can stabilize compact skyrmions in the absence of DMI, with sizes down to ~20 nm, despite frequent statements to the contrary^22,39,50^. On the other hand, stray field skyrmions with DMI below the transition line in Fig. 2c can have purely Néel domain walls, and possess either a compact or bubble-like profile (Fig. 2e). The profile can be continuously tuned from one to the other without changing the topology, either by applied field or by adjusting $\kappa =\frac{\pi {D}_{i}}{4\sqrt{A{K}_{u}}}$ (Fig. 2e, inset), as also shown in^34,35^. Hence, skyrmions with DMI-stabilized Néel domain walls are not necessarily DMI-stabilized skyrmions, even if their profiles are compact, since the energy giving rise to the minimum in *E*(*R*) can still derive from stray fields.

Based on Fig. 2c and the underlying energetics, we offer here mathematically-precise and experimentally-accessible criterion to categorize skyrmions unambiguously, defining the terms “DMI skyrmions”, “stray field skyrmions”, and “bubble skyrmions” as labeled in Fig. 2. The mathematical definition is based on the understanding of Fig. 2d. We first note that all contributions to the total energy can be classified into domain wall energies ($\propto \,R$ at large *R*) and bulk energies (all other energies), see also Figs 1c and d. Exchange, anisotropy, DMI, and volume stray field energies are domain wall energies. The Zeeman energy is a bulk energy. Surface stray fields contribute to both categories: the domain wall contribution leads to an effective reduction of the anisotropy and the bulk contribution effectively reduces the external field. We see that the minimum at small *R* is formed by the local energies while the minimum at large *R* is essentially a minimum in the bulk energies (which can be shifted due to the linear slope of the domain wall energies), see Fig. 2d. We therefore identify DMI skyrmions as minima of the domain wall energy. Stray fields may still play an important role in shaping this minimum and giving it the required depth for thermal stability, but the origin is still the minimum in the domain wall energy term that can only appear due to DMI. We include in this definition skyrmions whose domain wall energy has a negative slope at the minimum. By this definition, we include all skyrmions in materials with globally negative domain wall energy density. Physically, these states behave like minima in the domain wall energy, but they require a magnetic field to stabilize isolated skyrmions against expanding to helical states or skyrmion lattices. Finally, we call an energy minimum a “mixed state” if it is formed by minima of both domain wall and bulk energies (e.g., when both minima are at the same radius or when the energy barrier in between is so small (compared to *k*_*B*_*T*) that they form one extended stable state).

The domain wall energy minimum is the key aspect that the wall energy model neglects in its approximations. Its origin lies in “domain wall compression” wherein the reduction of Δ at small *R* leads to the zero radius energy of 27*Ad* (instead of 0 in the wall energy model). Domain wall compression leads to a steep increase of the domain wall energy (mostly exchange) at small *R*, which is why domain wall energy minima can be thermally stable down to much smaller radii than minima in bulk energies. This is illustrated in Fig. 1c. Even when considering that the ground state skyrmion energy already includes some compression energy, the difference between a linear interpolation of the domain wall energy according to the wall energy model and the correct result is 17*Ad* (27*Ad* instead of 10*Ad*). This additional 17*Ad* in stabilizing energy is one of the reasons why DMI skyrmions are physically different from stray field skyrmions.

**Figure 3:**
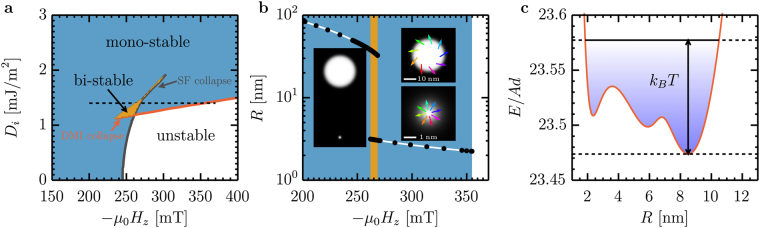
Multiple minima in systems with competing DMI and stray-field energies. (**a**) Phase diagram of the multi-stability of skyrmions as a function of applied field and DMI strength, where white, blue, and orange indicate the regions of instability, mono-stability, and bi-stability, respectively. Stability requirement is that all energy barriers are larger than 50*k*_*B*_*T*. The red line is the collapse field of DMI skyrmions and the gray line is the collapse field of stray field skyrmions. The dashed horizontal line indicates the slice that is plotted in panel (**b**). The data was obtained for a magnetic layer thickness *d* = 31.6 nm, a total film thickness of 4*d*, and magnetic layer *M*_*s*_ = 1.4 MA/m and quality factor *Q* = 1.01. (**b**) Radius as a function of applied field for skyrmions in the bi-stability region in (**a**) at *D*_*i*_ = 1.4 mJ/m^2^. The background color indicates the level of multi-stability as defined (**a**). The left inset shows a simulation of the stray field and DMI skyrmion which are simultaneously present in the same simulation area at *μ*_0_*H*_*z*_ = −254 mT. The right insets show zoom-ins on the individual skyrmions (stray field skyrmion on top, DMI skrymion at the bottom). The coloured arrows indicate the domain wall spin orientation. (**c**) Energy as a function of radius for a zero stiffness skyrmion. The thermal energy available to the skyrmion at room temperature is indicated by the blue shading and the dotted lines. All states between *R* = 2 nm and *R* = 11 nm are accessible by thermal excitation on a sub-nanosecond time scale. The shown data corresponds to *d* = 4 nm (no spacer layers), *D*_*i*_ = 1.85 mJ/m^2^, *Q* = 1.4, and *μ*_0_*H*_*z*_ = −100 mT. The minimum at $R\approx 6\,{\rm{nm}}$ is an artifact within the 1% precision of our model. The model actually predicts at most two minima.

Our mathematical definition is directly linked to discernibly different physical properties. First, the radius and in particular the collapse radii are different. Second, the collapse field shows distinct scaling: it is almost independent of DMI for stray field skyrmions and strongly increases with DMI for DMI skyrmions (see Fig. 3a). Third, Δ is determined by a competition between exchange and anisotropy for stray field skyrmions, but for DMI skyrmions it reflects a balance between DMI, anisotropy, and external field. That is, Δ is independent of *D*_*i*_ for stray field skyrmions and scales linearly with *D*_*i*_ for DMI skyrmions. Fourth, DMI skyrmions are rather insensitive to applied fields, while stray field skyrmions are quite sensitive. And fifth, *ψ* is variable for stray field skyrmions but not for DMI skyrmions.

The collapse radius is a particularly useful and technologically-relevant distinguishing feature. As shown in Fig. 2c, DMI skyrmions are those with room-temperature collapse diameters <10 nm and stray field skyrmions are those collapsing at larger sizes. This observation is supported by similar diagrams as a function of all material parameters, see Supplementary Information. With a very few exceptions (at small thicknesses), the 10 nm threshold is unambiguous even with some experimental error. That is, skyrmions either collapse at much larger or much smaller diameters than 10 nm. Even though further research is required to check if this threshold would change for other classes of materials (e.g., B20 materials or ferrimagnets) and for skyrmion lattices, we conclude that the 10 nm threshold size is a practical and valid criterion to identify the type of a skyrmion experimentally, at least for transition metal ferromagnet multilayers.

Finally, we remark that all room temperature isolated skyrmions observed so far^11,22–27,50–56^ should be classified as stray field skyrmions. Isolated DMI skyrmions have been observed at cryogenic temperatures^19,57,58^, but not yet at room temperature. The DMI strength measured in sputtered multilayers, however, can be on the order of 2 mJ/m^2^^22–24,59–63^, which we predict to be sufficient to stabilize DMI skyrmions. But the bi-stability region can make it difficult to obtain them by just shrinking stripe domains, in particular when pinning is sizable^56^. From our analysis is seems likely that DMI skyrmions can be observed in existing sputtered multilayer materials by using novel skyrmion generation approaches, such as spin-orbit torque nucleation^51^. To verify that these skyrmions are DMI skyrmions, one only needs to confirm that they can be as small as 10 nm in diameter.

### Bi-stabilities and zero stiffness skyrmions

The phase diagram of skyrmions in materials with strong stray fields shows a richness that has not been explored due to the inherent limitations of existing models. A particularly fascinating example is the appearance of multiple (possibly degenerate) minima in *E*(*R*). These minima can be separated by energy barriers exceeding 50*k*_*B*_*T*_RT_ at room temperature, indicating that both states can be simultaneously stable. Although the possible coexistence of compact and bubble-like has previously been suggested^39,49^, concrete model predictions of bi-stability have only been made in the case of systems stabilized by lateral constraints^64^. Our model shows that bistability is a more general phenomenon. As depicted in Fig. 3a, bi-stability exists in a small pocket of the phase diagram near the point where the collapse fields of stray field and DMI skyrmions coincide. The two types of skyrmions in the bi-stability region can have very different properties (Fig. 3b), confirmed by micromagnetic simulations: Their radii differ by more than one order of magnitude and their spin structure is Néel-like for the small (DMI) skyrmion and intermediate for the large (stray field) skyrmion. The different size and domain wall angle can be used to encode information or to move the skyrmions in non-collinear directions by spin orbit torques.

If the energy barrier between the multiple minima is on the order of *k*_*B*_*T* or smaller, thermal fluctuations can cause the system to dynamically oscillate between these minima with frequencies on the order of the attempt frequency (reported to be between 2.5 × 10^7^s^−1^ ^65^ and 4.5 × 10^−9^ s^−1^ ^47^). We therefore call these states zero stiffness skyrmions. Figure 3c shows *E*(*R*) for a system in which the radius can thermally fluctuate between 2 nm and 11 nm, such that it exhibits effectively zero stiffness with respect to variations of radius within this range. Zero stiffness skyrmions exists at larger DMI than bi-stable skyrmions (see Fig. 2d). We expect that zero stiffness skyrmions have a very low resonance frequency associated with their breathing mode, which could be exploited in nonlinear skyrmion resonators^66^ and should have impact on their inertia^11^ and on skyrmion Hall angle^52,53^. The ultra short timescale of sizable thermal fluctuations (a factor five in radius) and the resulting randomness of spin-orbit torque driven motion could also be used to implement high-speed skyrmion randomizers and stochastic computing^67^.

### Skyrmions for racetrack memory applications

**Figure 4:**
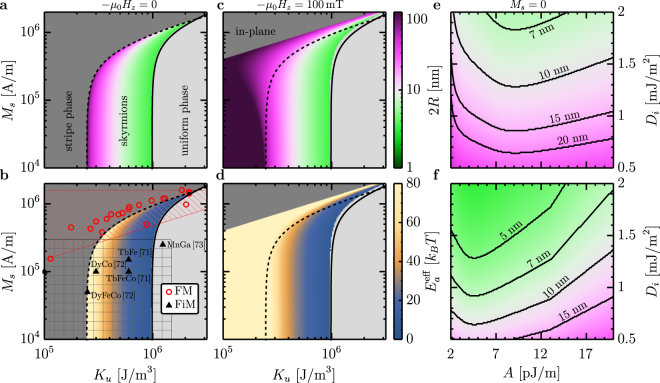
Zero field skyrmions. (**a**) Diameter of zero field skyrmions as a function of anisotropy and saturation magnetization, in a 2 nm thick film with *A* = 10 pJ/m and *D*_*i*_ = 2 mJ/m^2^. The black solid line is a plot of $\kappa =\frac{1}{2}$ and the dashed line follows the solution of $\sqrt{8A(Q-\mathrm{1)}{\mu }_{0}{M}_{s}^{2}}-\pi {D}_{i}={\mu }_{0}{M}_{s}^{2}d$, where the latter has been imposed as a threshold because stripe domains are expected to nucleate spontaneously if *K*_*u*_ is smaller^23^. (**b**) Stabilizing energy (in units of *k*_*B*_*T*) for the skyrmions in (**a**). The boxes illustrate the range of anisotropy and saturation magnetization values reported in the literature for different material classes. (**c,d**) The same diagrams as in (**a** and **b**), but for an applied field of 100 mT. (**e,f**) Diameter of zero-*M*_*s*_ skyrmions in a *d* = 5 nm (**g**) and a *d* = 10 nm (**h**) thick film as a function of exchange constant and DMI strength. The anisotropy constant at each point is adjusted to obtain ${E}_{a}^{{\rm{eff}}}=50{k}_{B}T$.

We now consider the design of skyrmions suitable for applications, such as racetrack-type memory devices in which bit sequences are encoded by the presence and absence of skyrmions that can be shifted by current^12–14,68^. Three necessary attributes are (i) small bit sizes, (ii) long term thermal stability, and (iii) stability in zero or low applied field. Figure 4 explains why ferromagnetic films and multilayers are incapable of meeting these requirements, and identifies alternative materials that can host sub-10 nm zero-field skyrmions at room temperature.

Zero field skyrmions reside at minima of the domain wall energy, with energy barriers ${E}_{a}^{0}$ and ${E}_{a}^{\infty }$ that prevent shrinking to zero size and infinite growth, respectively. We take the smaller of these as the effective energy barrier ${E}_{a}^{{\rm{eff}}}$ to estimate stability. Figures 4a,b show size and stability, respectively, for zero-field skyrmions as a function of *K*_*u*_ and *M*_*s*_ for thin films with *D*_*i*_ = 2 mJ/m^2^, typical of heavy-metal/ferromagnet interfaces. Stray fields tend to reduce ${E}_{a}^{\infty }$ and hence zero field skyrmions occur preferably at low *M*_*s*_, but there is an inherent tradeoff in Figs 4a,b between stability and size. Moderate applied fields (Figs 4c,d) extend the stability range for large stray field skyrmions but do little to stabilize small skyrmions.

Since it is the domain wall energy whose minimum is responsible for zero-field skyrmions, stability can be enhanced by increasing film thickness since this energy scales with *d*. For higher *M*_*s*_ materials, the benefit is offset by destabilizing stray fields that lead to stripe domain formation. But at low *M*_*s*_, sub-10 nm skyrmions can readily be achieved, as shown in Figs 4d,e for the limit *M*_*s*_ = 0. These figures show zero-field skyrmion size versus *A* and *D*_*i*_, with *K*_*u*_ adjusted at each point to ensure ${E}_{a}^{{\rm{eff}}}=50{k}_{B}{T}_{{\rm{RT}}}$. As a rule of thumb, *κ* ≈ 0.8 can be used to estimate *K*_*u*_ from the diagram.

Figure 4b shows parameter ranges for several classes of materials, providing guidance to realize such skyrmions experimentally. The problem with thin-film ferromagnets is that the quality factor $Q=\frac{2{K}_{u}}{{\mu }_{0}{M}_{s}^{2}}$ is usually constant when engineering multilayers or alloys, which correlates *K*_*u*_ and *M*_*s*_ in a way that makes the low-*M*_*s*_, high-*K*_*u*_ region inaccessible. This is reflected in the diagonal lower boundary marking the accessible parameter range for such materials (red box, Fig. 4b), and in the trend for experimental film parameters (red circles) reported in the literature. This explains why room-temperature skyrmions observed in ferromagnetic films and multilayers have all been quite large. Note that all previously observed isolated zero field skyrmions in Co-based ferromagnets are only stabilized by pinning or sample boundaries^23^.

Ferrimagnets, by contrast, generally show little correlation between *K*_*u*_ and *M*_*s*_, as seen in the representative literature values marked by triangles in Fig. 4b. In rare-earth-transition metal alloys, these parameters can be tuned independently by temperature or composition to achieve the combination of low-*M*_*s*_ and high *K*_*u*_ needed for stabilizing small, zero-field skyrmions. Moreover, the low *M*_*s*_ and bulk perpendicular anisotropy in such materials allows for thicker films with strong PMA, which reduces the *D*_*i*_ needed to stabilize small skyrmions (compare Figs 4e,f). Finally, as seen in Figs 4e,f, values for *A* between 4 pJ/m and 10 pJ/m are ideal, and in the rare-earth-transition-metal ferrimagnets the exchange constants are found to be just in this range. In addition to compensated ferrimagnets, natural antiferromagnets^69–73^ are quite promising for ultrasmall skyrmions, as are Co-based synthetic antiferromagnets (SAFs) that minimize stray fields, where heavy-metal interfaces can provide substantial *K*_*u*_ and *D*_*i*_.

### Spin-orbit torque driven motion of skyrmions

**Figure 5:**
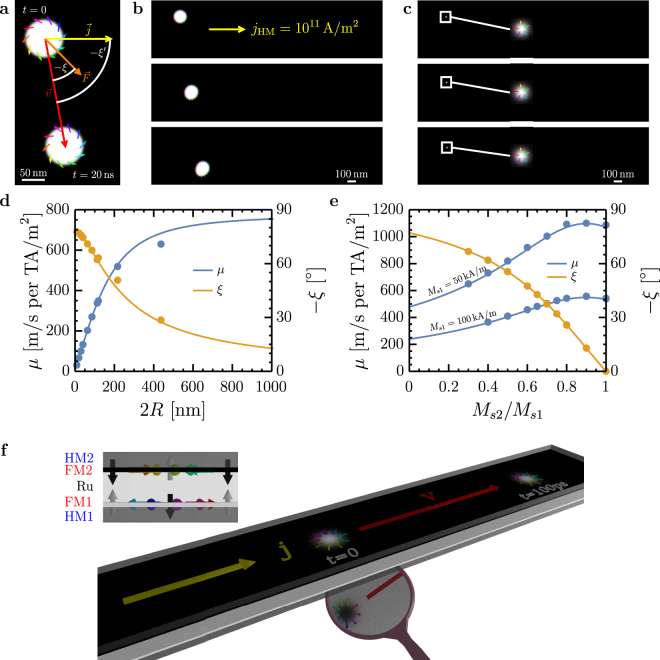
Spin-orbit torque driven skyrmion motion. (**a–c**) Simulated skyrmion motion for different ferromagnetic skyrmions, moved by a pure damping-like SOT current j_HM_ = 10^11^ A/m^2^. (**a**) Intermediate skyrmion with *ψ* ≈ 45°, illustrating the different directions of current density **j**, force **F**, and velocity **v** as well as the difference between *ξ* and *ξ*′. Other parameters are *M*_*s*_ = 1.4 MA/m, *Q* = 1.4, *A* = 10 pJ/m, *D*_*i*_ = 0.7 mJ/m^2^, ${\mathscr{N}}=10$ layers (affects the vertical spin current), *d* = 10 nm and total film thickness (including non-magnetic spacers) of 40 nm. (**b**) Motion of a 2*R* ≈ 200 nm diameter skyrmion at *μ*_0_*H*_*z*_ = −4.6 mT. (**c**) The same skyrmion as in (**b**), but at a field of *μ*_0_*H*_*z*_ = −90 mT and a corresponding diameter of 2*R* ≈ 20 nm. (**d**) Mobility (velocity per current density) and skyrmion Hall angle as a function of skyrmion diameter (the same skyrmion as in (**b**) and (**c**). The size is controlled by the out-of-plane field). Material parameters are *M*_*s*_ = 1MA/m, *K*_*u*_ = 765kJ/m^3^, *A* = 20 pJ/m, *D*_*i*_ = 2 mJ/m^2^, ${\mathscr{N}}=1$ layer, d = 1 nm without non-magnetic spacers. Simulations in (**d**) were performed using current densities between 10^11^A/m^2^ and 3 × 10^11^A/m^2^. (**e**) Mobility and skyrmion Hall angle for a SAF bilayer with fixed *M*_*s*1_ of the bottom layer and variable *M*_*s*2_ of the top layer. The same spin current is assumed for both layers. Other simulation parameters are *A*_*x*,*y*_ = 10 pJ/m, *A*_*z*_ = −10 pJ/m, *K*_*u*_ = 500 kJ/m^3^, *D*_*i*_ = 2 mJ/m^2^, and *d*_1_ = *d*_2_ = 2.5 nm. No field is applied. (**f**) 3D representation of a simulated 10 nm diameter skyrmion in a SAF bilayer, moving colinear with the current at a velocity of 1000 m/s at j_HM_ = 10^12^ A/m^2^, using *M*_*s*1_ = *M*_*s*2_ = 50 kA/m and other parameters as in (**e**). The two magnetic layers are colored black (top layer, predominantely magnetized down) and white (bottom layer, magnetized up). The skyrmion texture in the bottom layer can be seen in the reflection in mirror included in the ray-traced image. The small image shows a side view. Thermal stability of this skyrmion is 34*k*_*B*_*T*. In all simulations, *α* = 0.2 and ${\theta }_{{\rm{SH}}}^{{\rm{eff}}}=0.15$.

Finally, we derive analytical expressions for skyrmion dynamics that agree well with micromagnetic simulations and guide material selection to realize current-driven velocities exceeding 1000 m/s for sub-10 nm skyrmions. Figure 5a shows an intermediate skyrmion driven by damping-like spin-orbit torque (SOT) from a charge current density **j**_HM_ in an adjacent heavy metal. Due to its topological charge, the skyrmion moves at an angle *ξ*′ with respect to **j**_HM_, a phenomenon known as the skyrmion Hall effect^52,53^. For Néel skyrmions *ξ*′ coincides with the skyrmion Hall angle ξ, but in general *ξ* ≠ *ξ*′ since the current-induced effective force **F** depends on domain wall angle *ψ*.

Figures 5b,c show micromagnetic simulations of SOT-driven motion for a 200 nm and 20 nm skyrmion, respectively, in a ferromagnetic Co-based multilayer. The skyrmion Hall angle and current-driven mobility *μ* = *v*/*j*_HM_ are plotted versus skyrmion size in Fig. 5d. One sees that for small skyrmions *ξ* approaches *π*/2 and the mobility drops dramatically (linearly with radius) so that ultrasmall ferromagnetic skyrmions are effectively immobile. These phenomena have been noted in previous micromagnetic simulations^50^, but so far neither an intuitive explanation nor a materials-based solution for this technologically-critical challenge have been presented.

In the Supplementary Information we use the Thiele equation^74^ to derive the steady state velocity **v** = *v*(cos*ξ*′, sin*ξ*′) of a skyrmion driven by spin-transfer^75^ and spin-orbit torques based on the 360° DW model. Our treatment includes multilayers such as synthetic ferrimagnets or antiferromagnets with multiple sub-lattices *i* of variable *d*_*i*_ and *M*_*s*,*i*_, and can be extended to natural ferrimagnets by suitably scaling γ and α^83^. The solid lines in Fig. 5d,e show the results of our analytical model applied to ferromagnets and synthetic antiferromagnets, respectively. Although our model cannot account for internal deformations, we find excellent agreement with micromagnetic simulations for skyrmion diameters <200 nm, which distort minimally in micromagnetic simulations and are the most technologically relevant.

For damping-like SOT the angle between **v** and **j**_HM_ is

2 $${\xi }^{\text{'}}=\xi -\psi +\pi \mathrm{[1}-{\rm{\Theta }}({\theta }_{{\rm{SH}}}^{{\rm{eff}}}N)]$$

with

3 $$\xi ={\rm{a}}{\rm{t}}{\rm{a}}{\rm{n}}2(\mathop{g}\limits^{ \sim },\alpha ),$$

4 $$\tilde{g}=-\frac{4\langle N\rangle }{{I}_{A}(\rho )},$$

5 $${I}_{A}(\rho )=2\rho +\frac{2}{\rho }+1.93(\rho -0.65)\exp [-\mathrm{1.48(}\rho -\mathrm{0.65)}]\mathrm{.}$$

Here *α* is the Gilbert damping, ${\theta }_{{\rm{SH}}}^{{\rm{eff}}}$ is the effective spin Hall angle, and *I*_*A*_ is the exchange integral (see Supplementary Information). 〈*x*〉 denotes the average of *x* over all the layers or sublattices, weighted by *dM*_*s*_ of each layer: 〈*x*〉 = (∑_*i*_*d*_*i*_*M*_*s*,*i*_*x*)/(∑_*i*_*d*_*i*_*M*_*s*,*i*_). The mobility is given by:

6 $$\mu =\sum _{i}\frac{1}{\sqrt{\mathop{g}\limits^{ \sim }{(\rho )}^{2}+{{\alpha }_{i}}^{2}}}\frac{{\rm{\Delta }}{I}_{D}(\rho )}{{I}_{A}(\rho )}\frac{|{\theta }_{{\rm{S}}{\rm{H}},i}^{{\rm{e}}{\rm{f}}{\rm{f}}}|}{{d}_{i}{M}_{s,i}}\frac{{\gamma }_{i}\hslash }{2|e|}.$$

Here, the final term represents the summation of the current-induced effective fields in each layer, with *γ* the gyromagnetic ratio, *ħ* the reduced Planck constant and *e* the electron charge. The first term is a damping term that includes a topological contribution proportional to 〈*N*〉. For compact skyrmions, $|\tilde{g}(\rho )|\approx 0.9\gg \alpha$, i.e., the topological damping term dominates the dynamics. The ratio Δ*I*_*D*_(*ρ*)/*I*_*A*_(*ρ*) derives from the Thiele effective force, with

7 $${I}_{D}=\pi \rho +\frac{1}{2}\exp (-\rho )\mathrm{.}$$

For large *ρ* it is proportional to Δ, whereas for small *ρ* it is proportional to *R*, which explains why it is difficult to drive small skyrmions fast with SOT.

Based on these relations, there are only two ways to enhance the mobility of small, compact skyrmions: reduce the (topological) damping and enhance the current-induced effective field. The former can be accomplished using multi-sublattice materials and structures, such as ferrimagnets and SAF multilayers. The latter can be achieved using low-*M*_*s*_ materials interfaced with strong spin Hall metals, since the current-induced effective field scales as ${\theta }_{{\rm{SH}}}^{{\rm{eff}}}/{M}_{s}$, as verified experimentally for Ta/TbCo^76^.

Figure 5e shows *ξ* and *μ* for the bilayer SAF structure in Fig. 5f as a function of the compensation ratio *M*_*s*1_/*M*_*s*2_ of the antiferromagnetically-coupled layers, with parameters such that the layers host skyrmions with diameter of 10 nm. One finds an enhancement of the mobility as the system approaches magnetic compensation due to a reduction in the topological damping. Note that the mobility takes a maximum at ${M}_{s2}/{M}_{s1} < 1$ because *M*_*s*1_ is fixed in the simulations and *M*_*s*2_ is varied. Thus as *M*_*s*2_/*M*_*s*1_ decreases from unity, the average *M*_*s*_ also decreases, which initially leads to an increase of *μ*.

Prior micromagnetic simulations have suggested SAF structures to mitigate the skyrmion Hall effect by reducing the net topological charge. The corresponding reduction in topological damping is the main reason why larger skyrmion velocities are also observed^70,77^. We note that SOT driven domain walls in SAF structures have been shown to achieve enhanced mobility due to interlayer exchange torque^78^, a mechanism that is fundamentally different from what we identify here, and which is not effective for skyrmions due to the circular symmetry. Finally, Fig. 5e shows that by decreasing *M*_*s*_ of the constituent layers to also increase the spin Hall effective field per unit current density, velocities exceeding 1000 m/s can be achieved at a reasonable current density of 10^12^A/m^2^ for 10 nm skyrmions, as required for applications.

Based on the material requirements identified above, (i) a low *d*_*i*_*M*_*s*,*i*_ in each layer, (ii) a large effective spin Hall angle in each layer, (iii) two (or more) antiferromagnetically coupled layers or sub-lattices with a low (ideally zero) average topological charge 〈*N*〉, we can now identify particular materials. We predict that one of the most promising materials is Pt/GdCo/GdFe/Ir/Ta. GdCo/GdFe bilayers can be coupled antiferromagnetically by just tuning one of the layers to be above and one to be below the compensation temperature^79^. The antiferromagnetic coupling is much stronger than for RKKY coupled multilayers and not sensitive to the layer thicknesses. The material has low *M*_*s*_ and bulk perpendicular magnetic anisotropy, i.e., it can be easily grown to be 5 nm thick in a single layer. Pt and Ir lead to large additive DMI in combination with Co and Fe, respectively^22,59^. Finally, Pt and Ta are known to be efficient spin-orbit torque materials with opposite (additive) sign of the spin Hall angle.

## Discussion

In this article, we have presented the first unified theory that describes both stray field and DMI skyrmions in one coherent model. In order to make this model readily available, we have included software implementations in an online repository^84^.Thanks to the accurate expression of stray field interactions, we have been able to answer a number of fundamental physical questions, such as the meaning of topological stability and the proper and physically justified distinction of DMI and stray field skyrmions. Importantly, only DMI skyrmions can be <10 nm in diameter at room temperature. We have demonstrated that stray field interactions in ferromagnetic materials prevent the formation of such DMI skyrmions at at moderate applied fields and presently accessible values of *D*_*i*_ ≤ 2 mJ/m^2^ and that low-*M*_*s*_ materials are hence required to observe sub-10 nm skyrmions at room temperature. Furthermore, we find that small skyrmions in ferromagnets are much slower than in ferrimagnets or (synthetic) antiferromagnets. While this result is similar to regular domain walls, the underlying physics is different: while antiferromagnetic domain walls benefit from an exchange torque^78^, skyrmions instead are affected by a topological damping that can only be reduced in systems with antiferromagnetically coupled sublattices. Considering the topological damping is hence of crucial imporance for skyrmion-related materials science. It hence became clear that the future of skyrmionic lies in ferrimagnets and antiferromagnets and that transition metal ferromagnetic multilayers cannot be engineered to meet the requirements of data storage applications. Finally, we note that our model does not treat twisted states that could arise in low-DMI systems with strong stray field interactions^42^, nor does it treat skyrmions stabilized by more exotic phenomena such as frustrated exchange interactions^80–82^. However, the most suitable materials for applications are strong DMI systems with small, highly-mobile skyrmions, and our model accurately predicts all relevant physics for fundamental and applied studies of such skyrmions.

## Electronic supplementary material

Supplementary Information
