# Supplementary material for: Theory of isolated magnetic skyrmions: From fundamentals to room temperature applications
Source: Sci Rep. 2018 Mar 13;8:4464. doi: 10.1038/s41598-018-22242-8 (PMC5849609; doi:10.1038/s41598-018-22242-8)
Supplement: Supplementary file 1 — Supplementary Information [file 41598_2018_22242_MOESM1_ESM.pdf]

# Theory of isolated magnetic skyrmions: From fundamentals to room temperature applications

Felix Büttner*, Ivan Lemesh, and Geoffrey S. D. Beach

Department of Materials Science and Engineering, Massachusetts Institute of Technology, Cambridge, Massachusetts 02139, USA

February 5, 2018

## S1 Definition of multi-stability

The $E(R)$ graph can show a rich energy landscape with multiple minima and maxima in between. However, as illustrated in Figs. 2d and 3c of the main paper, not all minima correspond to individually stable states. In fact, transitions between minima are possible by thermal activation and sometimes even at zero temperature. Consider two minima $R_1$ and $R_2$, with energies $E_1$ and $E_2$ and a maximum with energy $E_m$ in between. The state $R_1$ can in principle transform into $R_2$ if $E_2 < E_1 + 50k_BT$, which means that the skyrmion can at maximum invest the thermal energy to climb up in the energy landscape. However, during the transition the skyrmion needs to pass the energy barrier defined by $E_m$ and that is only possible if $E_m < E_1 + 50k_BT + 2Ad$, where we applied that empirically skyrmions can "tunnel" through energy barriers of $2Ad$ in height by temporal deformations. If state $R_1$ can transform into state $R_2$ but not vice versa, because $R_2$ is much lower in energy, then we consider $R_1$ to be unstable and exclude it from the subsequent discussion. If, however, $R_2$ can transform back into $R_1$, and if both $R_1$ and $R_2$ are stable against annihilation or irreversible deformation into another state, then $R_1$ and $R_2$ form an extended range of stability which we call a *block*. A block can consist of

*felixbuettner@gmail.com

one or several minima and two blocks $b_1$ and $b_2$ are *distinct* if no minima of $b_1$ can transform into any minima of $b_2$ and vice versa. Finally, we call the $E(R)$ graph with all its minima and maxima $n$-stable if it contains $n$ distinct blocks.

# S2 Summary of the analytic energy functions

## S2.1 Analytic expression for the skyrmion structure

In general, a vector field of magnetic moments $\mathbf{m}(\mathbf{r})$ can be conveniently expressed by (position-dependent) spherical angles $\theta$ and $\psi$ via $\mathbf{m} = \sin(\theta)\cos(\varphi+\psi)\hat{\mathbf{e}}_x + \sin(\theta)\sin(\varphi+\psi)\hat{\mathbf{e}}_y + \cos(\theta)\hat{\mathbf{e}}_z = \sin(\theta)\cos(\psi)\hat{\mathbf{e}}_r + \sin(\theta)\sin(\psi)\hat{\mathbf{e}}_\varphi + \cos(\theta)\hat{\mathbf{e}}_z$, where $r, \varphi, z$ are cylindrical coordinates. At equilibrium and in the absence of in-plane fields the spin structure of a skyrmion is radially symmetric and we assume a constant profile along the out-of-plane direction $z$ and a constant angle $\psi$, i.e., $\theta = \theta(r, \rho_0)$ and $\psi = \text{const}$. Note that the validity of a constant profile along $z$ needs to be checked if $Q = \frac{2K_u}{\mu_0 M_s^2} < 1$ and $d > \Delta$ [1], where $\Delta$ is the domain wall width as defined below. The constant $\psi$ is called domain wall angle. Skyrmions with $\psi = 0$ (magnetic moments pointing outwards) and $\psi = \pi$ (magnetic moments pointing inwards) are called Néel skyrmions, whereas skyrmions with $\psi = \pi/2$ (magnetic moments rotating counter-clockwise when viewed from positive $z$ direction) and $\psi = 3\pi/2$ (magnetic moments rotating clockwise) are called Bloch skyrmions. Essentially, $\psi$ is the angle between the domain wall magnetic moments and the radial direction. The cross-sectional domain wall profile $\theta(r, \rho_0)$ has been experimentally determined to follow [2, 3]

$$\theta(r, R_0, \Delta) = \theta_{\mathrm{DW}}(r - R_0, \Delta) + \theta_{\mathrm{DW}}(r + R_0, \Delta) - (N+1)\pi/2 \tag{1}$$

$$\theta_{\mathrm{DW}}(r, \Delta) = 2\arctan\left(\exp\left(\frac{r}{\Delta}\right)\right) = \arcsin\left(\tanh\left(\frac{r}{\Delta}\right)\right) + \frac{\pi}{2} \tag{2}$$

where $R_0$, $\Delta$, and $N$ are parameters to describe the skyrmion size, the domain wall width, and the core polarity, respectively. It is convenient to introduce reduced variables $\varrho = r/\Delta$, $\rho_0 = R_0/\Delta$, in which the profile simplifies to

$$\theta(\varrho, \rho_0) = 2\left[\arctan\left(\exp\left(\varrho - \rho_0\right)\right) + \arctan\left(\exp\left(\varrho + \rho_0\right)\right)\right] - (N+1)\pi/2. \tag{3}$$

Here, we only consider skyrmions with defect-free domain walls. Hence, the topological charge

$$N = (4\pi)^{-1} \int \mathrm{d}x\mathrm{d}y\, \partial_x \mathbf{m} \times \partial_y \mathbf{m}) \cdot \mathbf{m}. \tag{4}$$

defines the skyrmion polarity. $N = 1$ if the magnetization in the skyrmion points up (as assumed throughout our calculations) and $N = -1$ if the core magnetization points down. Thus, the skyrmion structure is completely quantified by $R_0$, $\Delta$, $\psi$, and $N$.

It is not intuitive to visualize the quantity $R_0$ (or $\rho_0$) in a skyrmion domain wall profile. In contrast, the radius $R$ of the skyrmion, defined by the $m_z(R) = 0$ contour, is readily accessible from experimental or simulation images. There is a simple formula that relates the parameter $\rho_0$ and the reduced radius $\rho = R/\Delta$ of a skyrmion:

$$\rho = \operatorname{arcsinh}\left(\frac{e^{2\rho_0} + 1}{2e^{\rho_0}}\right). \tag{5}$$

In reverse, the parameter $\rho_0$ of a skyrmion with given reduced radius $\rho > \rho_{\mathrm{min}} = \operatorname{arcsinh}(1)$ can be calculated analytically via

$$\rho_0 = \ln\left(\sinh\rho + \sqrt{\sinh^2\rho - 1}\right). \tag{6}$$

Note the minimum value of $\rho$, indicating that the minimum skyrmion radius is determined by the domain wall width. In bubble skyrmions, Eq. (6) simplifies to $\rho \approx \rho_0$. In compact skyrmions, $\rho \approx \rho_{\mathrm{min}} + \frac{1}{3}\rho_0^2$ is a good approximation.

The total energy $E$ of a universal skyrmion can be written as a sum of exchange energy $E_A$, anisotropy energy $E_K$, DMI energy $E_D$, demagnetization energy $E_d$, and Zeeman energy $E_Z$:

$$E(R, \Delta, \psi, d, A, K_u, M_s, D_i, D_b, H_z) = E_A + E_K + E_D + E_d + E_Z \tag{7}$$

We will provide expressions of the individual terms in the subsequent sections. Detailed steps of how these expressions are derived can be found in section 11. Note that we use SI units throughout the paper.

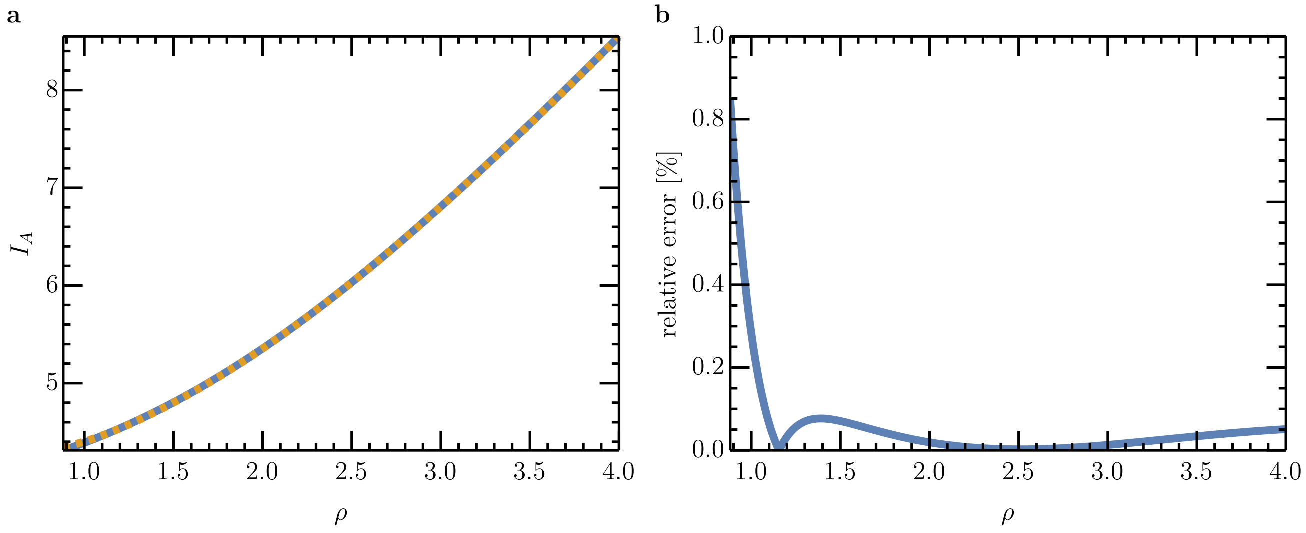


**Figure S1 | Exchange integral and analytic approximation.** **a**, Precise numeric evaluation of the exchange integral (solid blue line) and analytic approximation according to Eq. (8) (orange dashed line). **b**, Relative error of the analytic approximation according to Eq. (8).

## S2.2 Exchange energy

The exchange energy integral is given by $E_A = A \int r\mathrm{d}r\mathrm{d}z\mathrm{d}\varphi \left[(\partial_r\theta)^2 + r^{-2}\sin^2\theta\right] = 2\pi A d I_A$, which has no obvious analytical solution. However, numerical integration is straight forward and robust and we find an excellent approximation for all $\rho$ with relative error $< 1$ % given by

$$I_A \approx 2\rho + \frac{2}{\rho} + 1.93\,(\rho - 0.65)\exp\left[-1.48(\rho - 0.65)\right]. \tag{8}$$

The precision of the approximation is visualized in Fig. S1. For bubble skyrmions, Eq. 8 translates to $E_A \approx 4\pi A d R/\Delta$. For compact skyrmions, Eq. (8) has to be used. Note that Eq. (8) without the third term of the sum has been derived in earlier publications, but the result is not accurate in the limit of small $\rho$ [4]. In the limit $\rho \to \rho_{\mathrm{min}}$, the exchange energy is given by $E_A(\rho_{\mathrm{min}}) \approx 27Ad$, which is the only energy term that is finite in the limit of $R \to 0$. It does not diverge, though, leaving a finite energy barrier for the creation and annihilation of skyrmions even in the continuum model for the spin structure as used here.

## S2.3 Anisotropy energy

The integral $E_K = K_u \int_0^\infty \mathrm{d}^3r \sin^2\theta = 2\pi K_u d\Delta^2 I_K$ for the anisotropy energy can be solved analytically, with a complicated expression for $I_K$ as listed in section 11. However, as a function of $\rho$, $I_K$ is almost perfectly

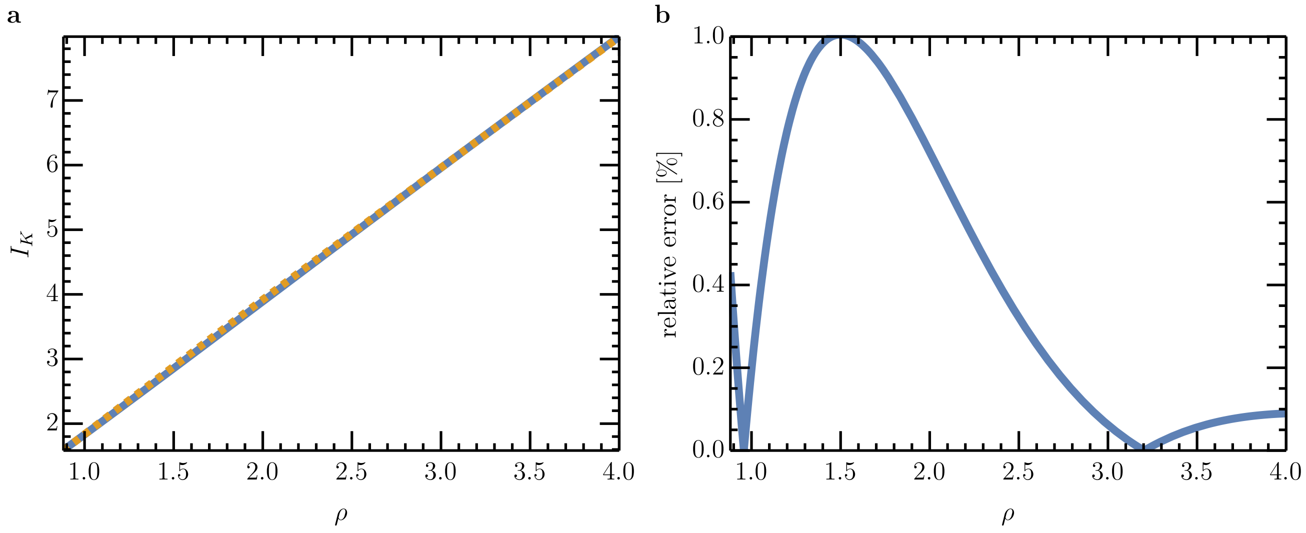


**Figure S2 | Anisotropy integral and analytic approximation.** **a**, Exact solution of the anisotropy integral (solid blue line) and simplified analytic approximation according to Eq. (9) (orange dashed line). **b**, Relative error of the simplified analytic approximation according to Eq. (9).

linear and can be approximated with less than 1 % error by the simple expression

$$I_K \approx 2\rho - \frac{1}{3}\exp\left(-\rho/\sqrt{2}\right), \tag{9}$$

as demonstrated in Fig. S2.

## S2.4 DMI energy

The DMI energy integral [5] $E_D = -2\pi d\Delta(D_i \cos\psi - D_b \sin\psi) I_D$ with $I_D = \int_0^\infty \mathrm{d}\varrho \left[\varrho\, \partial_\varrho \theta + \sin\theta \cos\theta\right]$ (with $\varrho = r/\Delta$) can also be solved analytically, with the lengthy result listed in section 11. Again, we can approximate the result with less than 1 % error for all $\rho$ by a much simpler function:

$$I_D \approx \pi\rho + \frac{1}{2}\exp(-\rho), \tag{10}$$

again as demonstrated in Fig. S3.

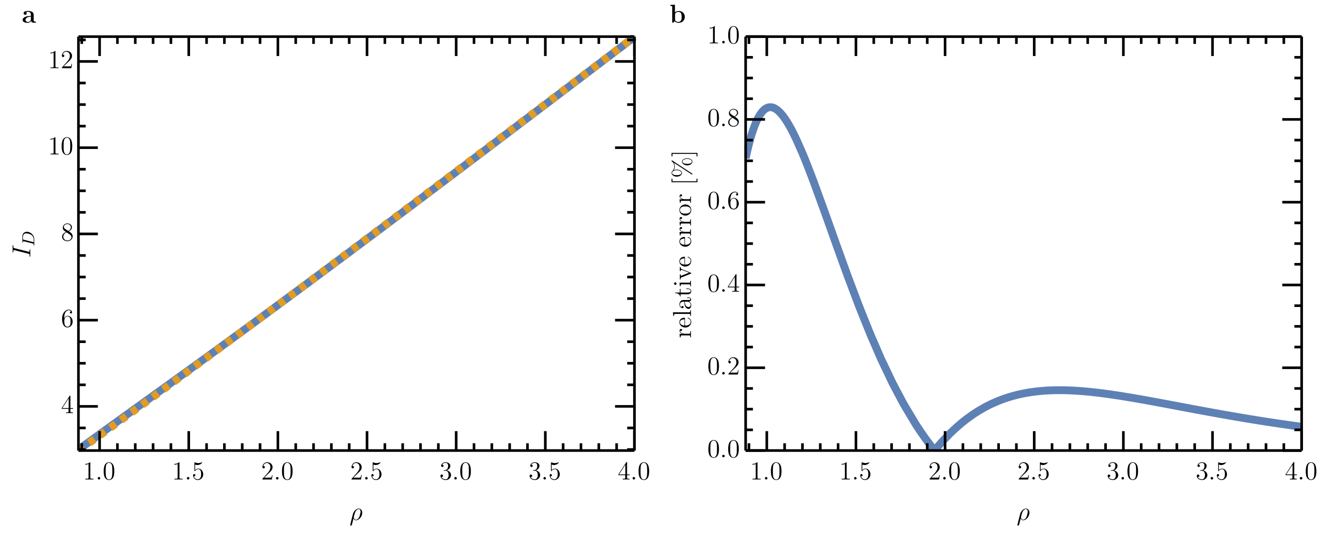


**Figure S3 | DMI integral and analytic approximation.** **a**, Exact solution of the DMI integral (solid blue line) and simplified analytic approximation according to Eq. (10) (orange dashed line). **b**, Relative error of the simplified analytic approximation according to Eq. (10).

## S2.5 Zeeman energy

The interaction with an external field is given by the integral $E_Z = -\mu_0 \int \mathrm{d}^3 r \mathbf{M} \cdot \mathbf{H}$. Assuming a pure out-of-plane field $H_z$, this simplifies to $E_Z = -2\pi\mu_0 M_s H_z d \Delta^2 I_Z$ with

$$I_Z \approx \rho^2 + \frac{\pi^2}{12} - 0.42 \exp(-\rho^2) \tag{11}$$

There is an exact analytic expression for $I_Z$ as listed in section 11. The approximation given here is accurate to less than 1 % and exact in the limit of large $\rho$, as one can see in Fig. S4. Note that a positive $H_z$ indicates that the field is aligned with the magnetization in the core of the skyrmion (thus favoring larger skyrmions). It may seem surprising that the Zeeman energy depends explicitly on $\Delta$ even for large radii. However, most other energies scale proportional to $R$ and the constant offset becomes insignificant for skyrmions with almost straight walls. Finally note that in the limit of large radii, the Zeeman energy converges to

$$E_Z(R \gg \Delta) = -\mu_0 M_s H_z V \tag{12}$$

with $V = 2\pi R^2 d$ being the volume of the skyrmion, as expected.

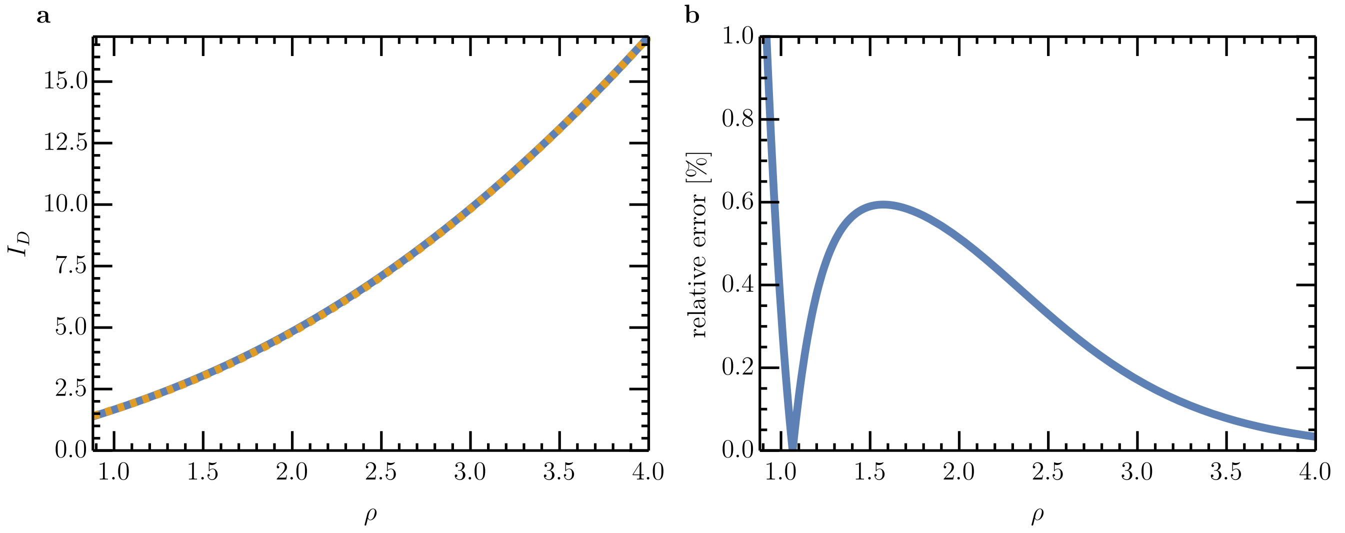


**Figure S4 | Zeeman integral and analytic approximation.** **a**, Exact solution of the Zeeman integral (solid blue line) and simplified analytic approximation according to Eq. (11) (orange dashed line). **b**, Relative error of the simplified analytic approximation according to Eq. (11).

## S2.6 Stray field energy

The stray field energy, demagnetization energy, or self energy, $E_d$ is half the Zeeman energy of the stray field $\mathbf{H}_s = M_s\mathbf{h}$

$$E_d = -\tfrac{1}{2}\mu_0 \int \mathrm{d}^3r\,(\mathbf{M}\cdot\mathbf{H}_s - M_s^2) = -\tfrac{1}{2}\mu_0 M_s^2 \int \mathrm{d}^3r\,(\mathbf{m}\cdot\mathbf{h} - 1) \tag{13}$$

where we have subtracted the stray field energy of a uniformly magnetized film (the $-1$ term in the integral) since we want to calculate the stray field energy of the skyrmion only [6].

There are two possible sources of stray fields: A divergent magnetization in the interior of the sample, causing so-called volume stray fields, and a magnetization component perpendicular to a sample surface, causing surface stray fields. That is, surface stray fields are created by the out-of-plane component $m_z$ and volume stray fields are due to the radial component $m_{\mathrm{ip}}$. Because of the assumed uniformity of $\mathbf{m}$ along the $z$ direction ($\mathbf{m}(z) = \mathbf{m}(-z)$) and the resulting symmetry $(\mathbf{h}_s)_{x,y}(z) = -(\mathbf{h}_s)_{x,y}(-z)$ of the in-plane components of surface stray fields, all interactions of surface stray fields with the in-plane components of the magnetization cancel in the $z$ integration. Similarly, volume stray fields do not interact with the out-of-plane magnetization and hence the total stray field energy is the sum of surface stray field energy $E_{d,s}$ and volume stray field energy $E_{d,v}$.

In the limit of ultra thin films, i.e., for $d \to 0$, the volume stray field energy is negligible (it scales with $d^2$ [7], whereas all other energy terms scale linearly with $d$) and the surface stray field inside the material simplifies to $h_z = m_z$ [8]. In that case, the integral in the stray field energy (13) is identical to the anisotropy energy integral and both can be combined using an effective anisotropy constant $K_{\text{eff}} = K - \frac{1}{2}\mu_0 M_s^2$. However, in reality, sample thicknesses are finite and to the best of our knowledge no estimate exists of how inaccurate the effective anisotropy model becomes at a given finite thickness. Subsequently, we will give an accurate approximation with error estimation of both surface and volume stray field energies for arbitrary sample thicknesses. Exact analytical expressions are provided for limiting cases.

## S2.7 Surface stray field energy

The surface stray field energy $E_{d,s} = -2\pi\mu_0 M_s^2 d\Delta^2 I_s$ of a circular symmetric structure involves highly non-trivial integrals in $I_s$, but numerical evaluations is possible, as detailed in section 11. Based on the numerical data, we find an analytical function for $I_s$ that approximates the correct result with less than 1 % error and becomes exact in limiting cases as discussed subsequently. In the limit of true two-dimensional films ($d \to 0$), the stray field acts as an effective anisotropy of strength $\frac{1}{2}\mu_0 M_s^2$,

$$\lim_{t\to 0} I_s = I_K, \tag{14}$$

whereas in the limit of very large thicknesses ($d \to \infty$), the surface stray field appears in the same form as a magnetic field of strength $M_s$ in the direction of the magnetization of the skyrmion core:

$$\lim_{t\to\infty} I_s = I_Z. \tag{15}$$

A formal proof of this identity is provided in section 11. For finite values of the film thickness, we observe a smooth transition between the two limiting cases of $d = 0$ and $d = \infty$ that resembles a tanh function on a semi logarithmic scale. The steepest slope of the transition function is around $d = R$, i.e., when the film thickness is equal to the skyrmion radius. The transition function for a cylindrical domain with a step-like domain wall profile, i.e., for $\Delta \to 0$, has been derived by Cape and Lehman [9] to be

$$E_{d,s}^0 = -2\pi\mu_0 M_s^2 dR^2 h(R/d) \tag{16}$$

with

$$h(x) = \frac{8x}{3\pi}\left[\frac{(1-k(x)^2)K(k(x)) + (2k^2-1)E(k(x))}{k(x)^3} - 1\right] \tag{17}$$

$$k(x) = \frac{2x}{\sqrt{1+4x^2}} \tag{18}$$

and

$$K(k) = \int_0^{\pi/2} \frac{\mathrm{d}x}{\sqrt{1-k^2\sin^2 x}}, \tag{19}$$

$$E(k) = \int_0^{\pi/2} \mathrm{d}x\sqrt{1-k^2\sin^2 x} \tag{20}$$

being the complete elliptic integrals of modulus $k$, which is tabulated in computer algebra software. Note that the elliptic integrals are not consistently defined in the literature and in software like Mathematica.

The total surface stray field energy of a skyrmion can be written as a sum of the zero wall energy $E^0_{d,s}$ and a contribution due to the finite wall width $E^\Delta_{d,s}$. For large skyrmion radii, $E^\Delta_{d,s}$ is expected to be equal to $2\pi Rd\sigma^\Delta_{d,s}$, where $\sigma^\Delta_{d,s} = \tilde{\sigma}^\Delta_{d,s}\mu_0 M_s^2\Delta$ with

$$\tilde{\sigma}^\Delta_{d,s} = \frac{\left[t + 2t\ln(\pi) - 2t\ln(t) + 4\pi\ln\left(\Gamma\left(\frac{t}{\pi}\right)\right) + 2\pi\right] - 4\pi^2 t^{-1}\psi^{(-2)}\left(\frac{t}{\pi}\right)}{2\pi} \tag{21}$$

is the normalized wall surface stray field energy of an isolated straight domain wall, which is generally negative between −1 for zero thickness and 0 for infinite thickness [10]. The symbols are $t = d/\Delta$, $\Gamma$ is the gamma function, and $\psi^{-2}(z) = \int_0^z \mathrm{d}t \ln\Gamma(t)$ is the second anti-derivative of the digamma function. Indeed, the expectation that the stray field energy is described by this wall energy model is in excellent agreement with the numerical results. For smaller radii, the coefficient $2\pi R$ needs to be replaced by $\pi\Delta I_K$ and a phenomenological correction term needs to be added to obtain a universal equation with less than 1 % deviation. The result reads

$$I_s(R,\Delta,d) \approx I_Z h(R/d) - \tfrac{1}{2}I_K\tilde{\sigma}^\Delta_{d,s}(t) - \frac{0.29 - 0.4\exp(-2\rho)}{\cosh^2\left(\frac{4}{9}\ln\left(\frac{d}{1.65R}\right)\right)}. \tag{22}$$

The error is indeed $< 1$ % if we use the exact expressions for $I_Z$ and $I_K$. However, we prefer to use the simple and robust approximations for those two integrals, as discussed in sections 2.3 and 2.5. The errors of the

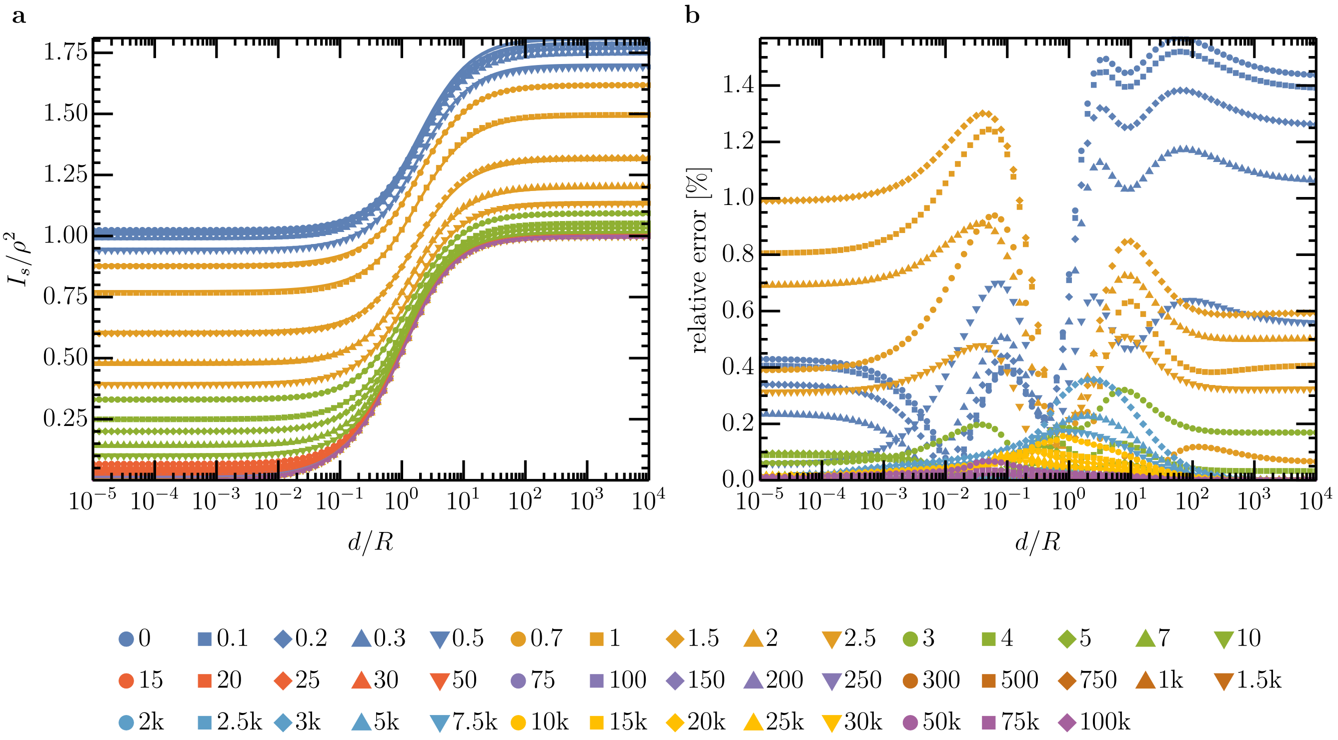


**Figure S5 | Surface stray field integral and analytic approximation.** **a**, Precise numeric solution of the surface stray field integral (dots) and analytic approximation according to Eq. (22) (lines). The symbol shape and color indicates different values of $\rho_0$, as defined in the legend below the plot. **b**, Relative error of the analytic approximation according to Eq. (22). The only data points with relative error $> 1\,\%$ all correspond to values of $\rho < 2$. Note that the error for some values of $\rho_0$ is so small that the corresponding symbols are not visible in the plot.

approximations then add up and relative error increases beyond 1 % As we show in Fig. S5, the total error is still smaller than 1.5 %). The simple approximation is therefore still sufficiently accurate to derive skyrmion properties from the total energy functional.

## S2.8 Volume stray field energy

As detailed in section 11, the volume stray field energy can be calculated via

$$E_{d,v} = 4\pi\mu_0 M_s^2 \Delta R d \cos^2(\psi) I_v, \tag{23}$$

where we evaluated $I_v$ numerically for a large variety of combinations of $R$, $d$, and $\Delta$. We find that the following function $I_v$, which is illustrated in Fig. S6, approximates the numerical results with less than 1 %

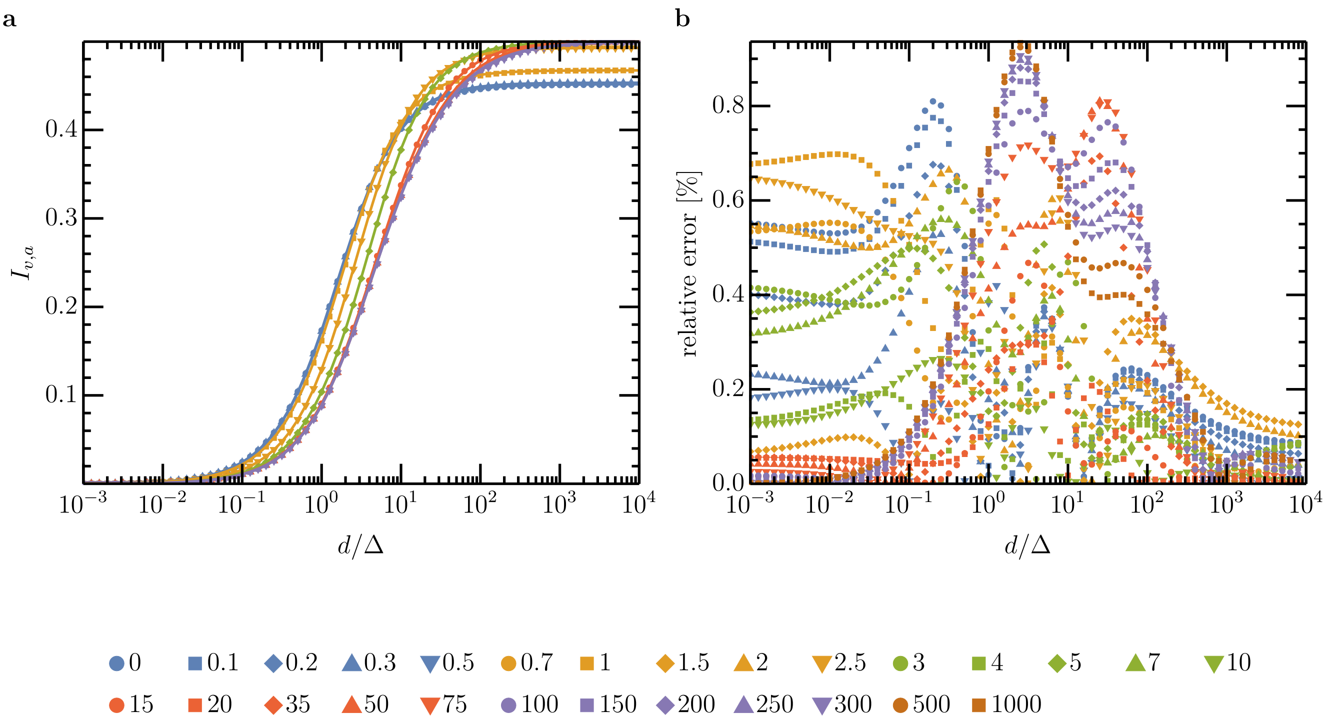


**Figure S6 | Volume stray field integral and analytic approximation.** **a**, Precise numeric solution of the volume stray field integral (symbols) and analytic approximation according to Eq. (24) (lines). The symbol shape and color indicates different values of $\rho_0$, as defined in the legend below the plot. To avoid overlapping, only every third value of $\rho_0$ is shown. **b**, Relative error of the analytic approximation according to Eq. (24) for all values of $\rho_0$.

relative error for all $R$, $\Delta$, and $d$:

$$I_{v,a}(\rho,t) = I_{v,a}(\rho,\infty)\frac{\ln(2)}{\pi} f_v\left(\rho, t\, a(\rho)\right) \tag{24}$$

with

$$f_v(\rho,t) = \frac{2\pi t}{\left((2\pi)^{n(\rho)} + (2t\ln(2))^{n(\rho)}\right)^{\frac{1}{n(\rho)}}} - \frac{t^2 10^{-0.02k(\rho)^2+1.3k(\rho)-2.45}}{(2t\ln(2)+3\pi)^{k(\rho)}} \tag{25}$$

$$n(\rho) = \frac{1}{6}\rho^{2/3}\exp(-0.31\rho) + 1 \tag{26}$$

$$k(\rho) = \frac{2.7 + \frac{14}{\rho-0.8}}{\exp(25-10\rho)+1} + \frac{10}{\exp(10\rho-25)+1} \tag{27}$$

$$\partial_t I_{v,a}(\rho,0) = \frac{4.45(\rho-0.283)}{\rho(\rho+0.93)^2} + \ln(2) \tag{28}$$

$$I_{v,a}(\rho,\infty) = \frac{1}{2}(1 - 0.27\exp(-1.164\rho)) \tag{29}$$

$$a(\rho) = \frac{\partial_t I_{v,a}(\rho,0)}{2\ln(2) I_{v,a}(\rho,\infty)} \tag{30}$$

We can understand this function as follows. Overall, $I_v$ mostly follows the theoretical prediction for the transverse anisotropy constant of a straight domain wall [7], with a constant value for large $t$, a linear dependence on $t$ for small $t$, and a transition approximately between $t = 1$ and $t = 10$. This straight wall function is well approximated by $\frac{\ln(2)}{2\pi} f_v$ with $n = 1$ and $k = 2.7$. For small $\rho$, however, we observe a stretching of the straight wall function along both axes of the plot. This is accounted for by the stretching factors $a$ and $I_{v,\mathrm{inf}}$. Finally, the transition between the linear and the constant regime is a function of $\rho$, which is accounted for by the dynamic exponents $n(\rho)$ and $k(\rho)$.

# S3 Scaling laws

The energy functional is invariant under the following two-dimensional transformatation with independent scalar variables $x$ and $y$:

$$d \to dx, \quad A \to Ay, \quad K_u \to K_u y/x^2 \tag{31}$$

$$D \to Dy/x, \quad M_s \to M_s\sqrt{y}/x, \quad H_z \to H_z\sqrt{y}/x. \tag{32}$$

This transformation results in

$$R \to Rx, \quad \Delta \to \Delta x, \quad \psi \to \psi, \quad E \to Exy. \tag{33}$$

For example, one can reduce the skyrmion size by a factor two at the same stabilizing energy by setting $x = 1/2$ and $y = 2$, i.e., by reducing the thickness and the anisotropy by a factor two while simultaneously increasing the exchange stiffness by a factor two, keeping the DMI fixed and reducing $M_s$ and $H_z$ each by a factor $1/\sqrt{2}$.

# S4 Domain wall angle

Consider a material with pure interfacial DMI (i.e., no bulk DMI). Under this constraint, $\psi$ can be obtained analytically via separation of variables:

$$\cos(\psi) = -f_{mm}\left(\frac{D_i I_D}{4\mu_0 M_s^2 R I_v}\right), \tag{34}$$

$$f_{mm}(x) = \begin{cases} \text{sign}(x), & |x| > 1 \\ x, & \text{else.} \end{cases} \tag{35}$$

We can now define a critical DMI strength $D_{c\psi}$ above which the skyrmion is of Néel type ($|\cos(\psi)| = 1$). Clearly, $\psi$ and $D_{c\psi}$ are functions of the skyrmion radius and not just based on material parameters. Indeed, as shown in the main paper, skyrmions with different DW angle can co-exist in the same material. However, we find empirically that the DW is more Néel-like for smaller skyrmions and that all skyrmions are of Néel type if the DMI value of the material exceeds the critical DMI $D_{c\psi}^{\mathrm{SW}}$ for isolated stripe DWs [10].

# S5 Behavior at large radii

At large $\rho \to \infty$, the total energy simplifies significantly. It is straight forward to show that in this limit, the so-called wall energy model, the total energy $E_{\mathrm{WEM}}$ reads

$$E_{\mathrm{WEM}} = 2\pi d\sigma_{\mathrm{DW}} R - \mu_0 M_s^2 d^2 R\,[6\ln(2) - 1 + 2\ln(R/d)] - 2\pi d\mu_0 M_s H_z R^2 \tag{36}$$

$$= 2\pi d\sigma_{\mathrm{DW}} R + aR - bR\ln(R/d) + cR^2 \tag{37}$$

with

$$\sigma_{\rm DW} = \frac{2A}{\Delta} + 2K_u\Delta - \pi(D_i\cos\psi - D_b\sin\psi) + \sigma^{\Delta}_{d,s}(d/\Delta) \tag{38}$$

$$+ \frac{1}{\pi}\ln(2)\mu_0 M_s^2\Delta\cos^2\psi\left[\frac{2\pi d/\Delta}{2\pi + 2\ln(2)d/\Delta} - \frac{8.2(d/\Delta)^2}{(2\ln(2)d/\Delta + 3\pi)^{2.7}}\right], \tag{39}$$

$$a = -\mu_0 M_s^2 d^2\,[6\ln(2) - 1], \tag{40}$$

$$b = 2\mu_0 M_s^2 d^2, \tag{41}$$

$$c = -2\pi d\mu_0 M_s H_z. \tag{42}$$

The condition “large radius” is met if $R > 10d$ and $R > 100\Delta$. For a given radius $R$, the field $\mu_0 H_z$ leading to a vanishing derivative $\partial_R E_{\rm WEM}$ can be calculated analytically:

$$\mu_0 H_z(R) = -\frac{b\ln(R/d) + b - a - 2\pi d\sigma_{\rm DW}}{4\pi R d M_s}. \tag{43}$$

This combination of $R$ and $H_z$ defines a minimum of $E_{\rm WEM}$ if $R$ is larger than the minimum radius of this model:

$$R > R_{\rm min,WEM} = d\exp\left(\frac{a + 2\pi d\sigma_{\rm DW}}{b}\right). \tag{44}$$

To find the minima of the total energy, we evaluate $E_{\rm WEM}$ for $R > \max(10d, 100\Delta)$ and the accurate energy model else.

# S6 Minimum radius $R_c$

The 360° domain wall model constrains $\Delta$ to values smaller than $R/\mathrm{arcsinh}(1)$. If for a given radius $R$ there is no energy minimum for $\Delta$ in $[0, R/\mathrm{arcsinh}(1)]$ then this radius cannot be stable. Therefore, we can define $R_c$ as the smallest possible radius for which $E(\Delta)$ has a minimum in $\Delta$ in $[0, R/\mathrm{arcsinh}(1)]$. The concept is illustrated in Fig. S7 for the example of $M_s = 1.4\times10^6$ A/m, $A = 10$ pJ/m, $Q = \frac{2K_u}{\mu_0 M_s^2} = 1.01$, $D_i = 3.2$ mJ/m$^3$, $\mu_0 H_z = -21$ mT, $d = 3$ nm (magnetic material thickness), and a total film thickness (magnetic material plus spacer layers) of 12 nm. For $R = 20$ nm, $E(\Delta)$ has a clear minimum at $\Delta = 7$ nm. For $R = 11$ nm, the minimum is very shallow but still present at $\Delta = 7.3$ nm (note the counter-intuitive fact that $\Delta$

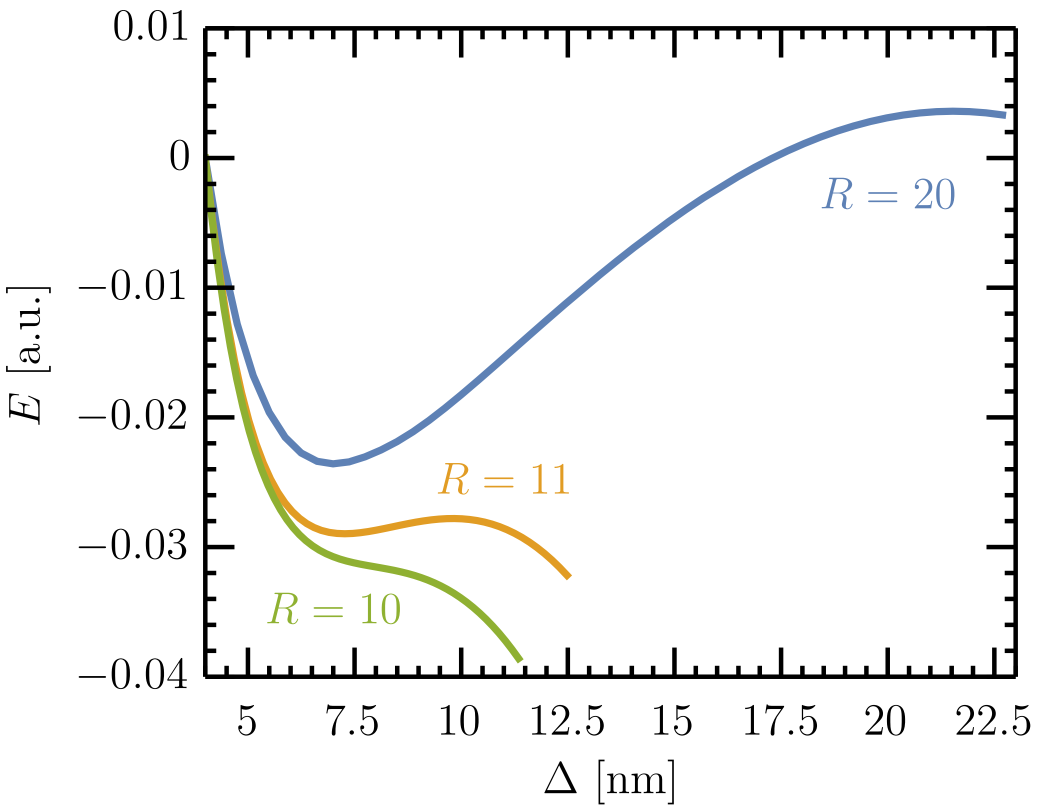


**Figure S7 | Minimum radius $R_c$.** Energy as a function of $\Delta$ for different fixed radii. Energies were scaled to fit in the same plot range. The curves end at $\Delta = R/\text{arcsinh}(1)$. For $R < 10.4$, $E(\Delta)$ does not have a minimum.

increases with decreasing $R$). At slightly smaller $R$, the minimum disappears and $E(\Delta)$ has a negative slope everywhere. The largest $R$ where $E(\Delta)$ still has a minimum is $R_c$. In the present example, $R_c = 10.4$ nm. Finally, note that the parameter set in the example is quite extreme to illustrate the effect. In most cases (i.e., larger anisotropy), $R_c$ is on the order of 1 nm and therefore coincides with the general limit of the continuous micromagnetic model.

## S7 Bogdanov and Hubert's effective anisotropy model

Here we briefly show how Bogdanov and Hubert's effective anisotropy skyrmion model [5, 11] can lead to explicit solutions when applying it to the 360° DW profile. Essentially, the effective anisotropy model approximates the magnetization-pattern-dependent surface and volume stray fields by material-dependent anisotropy terms $K_s$ and $K_v$, respectively. In the most sophisticated form, $K_v = K_\perp \cos^2 \psi$ depends on the domain wall angle (and actually also on $\Delta$ [7], but including such a level of complexity would bring us back

to our model).

If we insert the effective anisotropy simplification of the stray field energies into our original model, we obtain

$$\tilde{E} = \frac{E}{2\pi d} \tag{45}$$

$$= AI_A(\rho) - \Delta(D_i \cos\psi - D_b \sin\psi) I_D(\rho) + \Delta^2 \left[ K I_K(\rho) - \mu_0 M_s H_z I_Z(\rho) \right] \tag{46}$$

with $K = K_u + K_s + K_\perp \cos^2\psi$. It is straight forward to show the only simultaneous solution of $\partial_\Delta \tilde{E} = 0$ and $\partial_\psi \tilde{E} = 0$ for interfacial DMI ($D_b = 0$) is $\sin\psi = 0$, that is, a pure Néel type wall. That is, skyrmions in the effective anisotropy approximation cannot have a transient-type spin structure, in contrast to straight domain walls in the same material. With this information in mind, $\Delta$ can be analytically obtained as the solution of $\partial_\Delta \tilde{E} = 0$:

$$\Delta = \frac{|D_i| I_D(\rho)}{2K I_K(\rho) - 2\mu_0 M_s H_z I_Z(\rho)} \tag{47}$$

and the energy reduces to

$$\tilde{E} = AI_A(\rho) - \frac{D_i^2 I_D(\rho)^2}{4K I_K(\rho) - 4\mu_0 M_s H_z I_Z(\rho)}. \tag{48}$$

In zero field, the energy becomes a function of $\rho$ and $\kappa = \frac{\pi D_i}{4\sqrt{AK}}$ alone:

$$\frac{\tilde{E}(H_z = 0)}{A} = I_A(\rho) - \frac{4\kappa^2}{\pi^2} \frac{I_D(\rho)}{I_K(\rho)}. \tag{49}$$

Minimizing these energies (with and without field) is a simple numerical task, see Fig. S8. It is worth noting the completely different scaling of the domain wall width compared to the wall energy model: In the wall energy model, $\Delta = \sqrt{A/K}$ is formed by competition between exchange and anisotropy, while in Bogdanov and Hubert's model $\Delta$ is determined by the competition of DMI versus anisotropy and external field.

Estimating the effective anisotropy $K$ is not trivial because the stray field contribution scales with $\Delta$ (and $\Delta$ is a function of $K$). This complexity is properly treated in our model, which is essentially the key novelty. In the simple picture, one typically operates in one of two limits: The thin film limit ($K = K_u - \frac{1}{2}\mu_0 M_s^2$) and

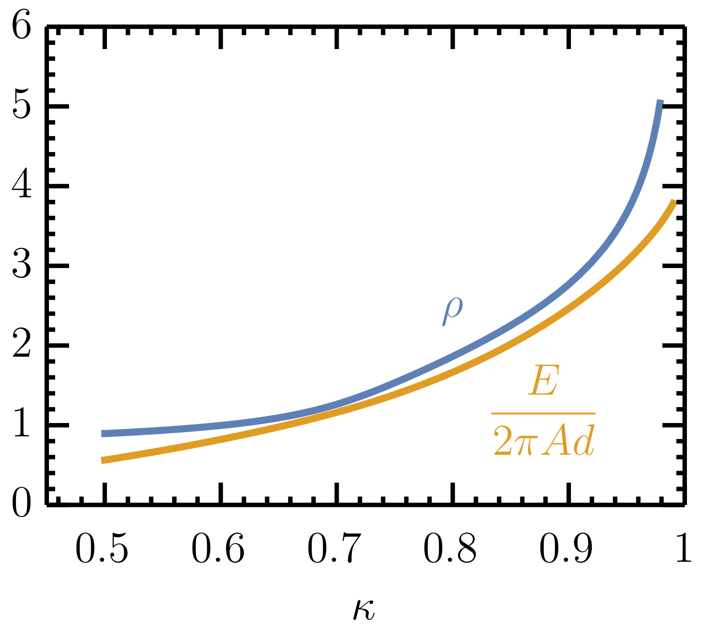


**Figure S8 | Solution of the effective anisotropy model in zero field.** For $\kappa < 0.5$ the 360° DW model fails to approximate the true solution. Both energy and radius diverge at $\kappa = 1$. The presented graph was calculated up to $\kappa = 0.99$.

the thick film limit ($K = K_u + \frac{1}{2}\mu_0 M_s^2 \cos^2\psi$). For our analysis, the thin film limit is more appropriate. This limit has been applied to generate Fig. 2b in the main text.

## S8 Collapse diameter for various material combinations

We have used our analytical model to derive the smallest stable room temperature size for a variety of material parameters, similar to the data shown in Fig. 2c of the main paper. The result is presented in Fig. S9. In the top row we vary the anisotropy and in the second and third row we vary $A$ and $M_s$, respectively, at constant $Q = 1.4$. In all cases we plot the smallest size as a function of DMI and magnetic film thickness in a multilayer material with total film thickness of $4d$. Note that the smallest radius is not necessarily obtained at the largest field at which skyrmions are stable, see discussion in the main text.

We first observe that the phase boundary between DMI skyrmions and stray field skyrmions is in all cases well described by the 10 nm diameter contour. Second, we see that the DMI required for a DMI skyrmion decreases with film thickness in all cases. At several tens of nanometer magnetic material thickness we often encounter DMI skyrmions at $D_i < 2\,\mathrm{mJ/m^2}$ and at fields on the order of 100 mT. Note that these skyrmions are much smaller than the film thickness. We cannot predict how twisting affects these states, i.e., non-uniform

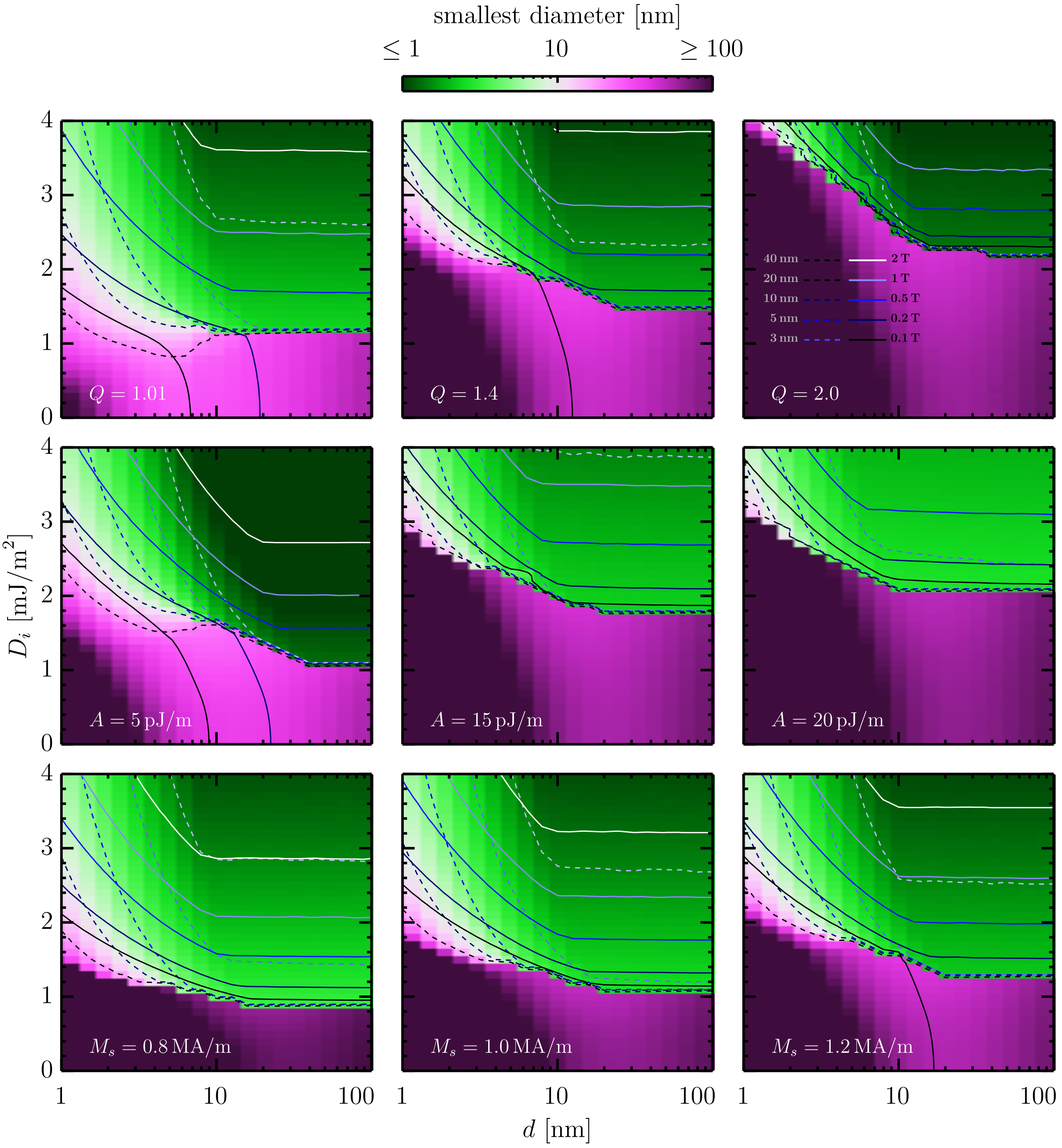


**Figure S9 | Smallest skyrmion diameter for various material combinations.** The color encodes the smallest possible size at the given material parameters. The dashed lines are contours of specific diameters as indicated in the legend in the top-right panel. The solid lines indicate the magnitude of the applied field required to obtain the displayed state.

| Number in Fig. S10 | Material | $K_u$ [kJ/m$^3$] | $M_s$ [kA/m] | Reference |
|---|---|---|---|---|
| 1 | $[CoFe/Pd]_n$ | 114 | 155 | 12 |
| 2 | CoCr | 177 | 450 | 13 |
| 3 | $[Pt/CoFeB/MgO]_n$ | 267 | 430 | 14 |
| 4 | oxidized $Co_2FeAl$ | 300 | 550 | 15 |
| 5 | CoPt | 341 | 380 | 16 |
| 6 | $[Pt/Co/Ta]_n$ | 376 | 600 | 14 |
| 7 | Ta/CoFe/MgO | 413 | 700 | 17 |
| 8 | Pt/CoFe/MgO | 483 | 700 | 17 |
| 9 | $Co_2FeAl$ | 526 | 731 | 18 |
| 10 | $[Ni/Co]_n$ | 600 | 835 | 19 |
| 11 | Cr/Fe/MgO | 609 | 900 | 20 |
| 12 | $[Ir/Co/Pt]_n$ | 749 | 960 | 21 |
| 13 | $[CoPt/TiN]_n$ | 877 | 500 | 22 |
| 14 | $[Pt/CoFeB/MgO]_n$ | 978 | 1120 | 23 |
| 15 | W/Hf/CoFeB/MgO | 1255 | 1200 | 24 |
| 16 | Ta/CoFeB/MgO | 1305 | 1200 | 25 |
| 17 | $[Co/Pt]_n$ | 1858 | 1600 | 21 |
| 18 | Fe/MgO | 2033 | 980 | 26 |
| 19 | Mo/CoFeB/MgO | 2164 | 1500 | 27 |
| 20 | $[Pd/Co]_n$ | 2171 | 1422 | 28 |
| 21 | FePt/FeCo | 7482 | 1250 | 29 |

**Table S1** | Ferromagnetic multilayers (labeled with $[]_n$) and alloys with perpendicular magnetic anisotropy. Some authors provide $K_u$ and $M_s$ normalized to the total film thickness (effective medium parameters) while others normalize to just the thickness of the magnetic material. As evidenced in Fig. S10, none of these values are within the stability regime for zero field skyrmions.

magnetization along the out-of-plane direction. We expect twisting at least near the surface of the film, where flux closure domains want to form. Intuitively, we expect that these twisted states are less stable than the uniform states described by our model, but it remains to be seen experimentally if DMI skyrmions can still exist in these thicker multilayers. The graphs presented in Fig. S9 indicate where such DMI skyrmions can be expected.

# S9 Materials for zero field skyrmions

In Fig. 4b of the main text, we have included literature values for a variety of different ferromagnetic and ferrimagnetic materials. Here, we list in more detail what these materials are and which references they were taken from.

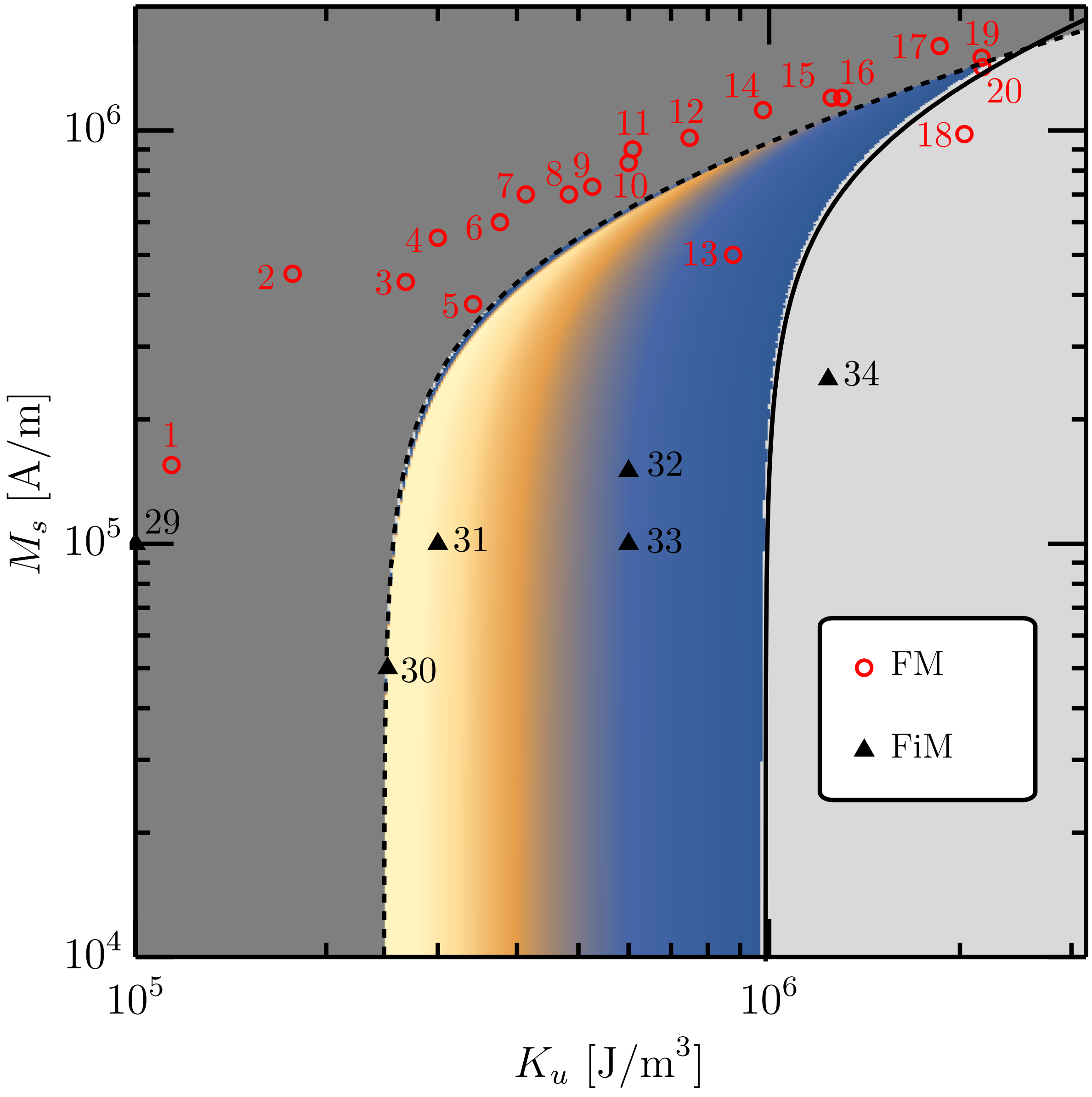


**Figure S10 | Stability of zero field skyrmions and map of material parameters of ferromagnetic and ferrimagnetic materials.** Reproduction of Fig. 4b from the main text with labeled literature data points. The numbers are explained in Tables S1 and S2.

| Number in Fig. S10 | Material | $K_u$ [kJ/m$^3$] | $M_s$ [kA/m] | Reference |
|---|---|---|---|---|
| 22 | HoFe | 0 | 50 | 30 |
| 23 | HoCo | 20 | 100 | 30 |
| 24 | GdFe | 40 | 500 | 31 |
| 25 | GdFeCo | 44 | 100 | 32 |
| 26 | GdCo | 50 | 100 | 33 |
| 27 | TbCo | 64 | 200 | 31, 34 |
| 28 | Al/TbFeCo/Al | 75 | 300 | 35 |
| 29 | DyFe | 100 | 100 | 30 |
| 30 | DyFeCo | 250 | 50 | 30 |
| 31 | DyCo | 300 | 100 | 30 |
| 32 | TbFe | 600 | 150 | 31 |
| 33 | TbFeCo | 600 | 100 | 31 |
| 34 | Cr/MgO/$Mn_{2.5}Ga$ | 1239 | 250 | 36 |

**Table S2 |** Ferrimagnetic materials with perpendicular magnetic anisotropy. Values of $K_u$ and $M_s$ typically depend on temperature and exact composition. Listed are room temperature values corresponding to the composition with largest $K_u$.

# S10 Spin orbit torques, skyrmion Hall effect, and dissipation

Spin orbit torques (SOT) describe the phenomenon that a current in a heavy metal, such as platinum, can lead to a perpendicular spin current with transverse polarization, as sketched in Fig. S11, and that this current can manipulate the magnetization of a ferromagnet on top of the heavy metal [37]. Following the example of Fig. S11, an electrical current in $x$ direction in a Pt layer creates an accumulation of magnetic moments polarized in positive $y$ direction at the top surface and in negative $y$ direction at the bottom surface. This is effectively a spin current $\mathbf{j}_s = (0, 0, j_s)$ because the spins are separated in $z$ direction (commonly referred to as the perpendicular direction). The spins themselves are polarized in $y$ direction, which is commonly called the transverse direction. In addition, there are adiabatic and non-adiabatic spin transfer torques due to spin-polarized current flow in the magnetic material [38]. The dynamics induced by these currents on skyrmions is described by the Landau-Lifshitz-Gilbert-Slonczewski equation [39–41]

$$\dot{\mathbf{m}} = -\gamma\mu_0\mathbf{m}\times\mathbf{h} + \alpha\mathbf{m}\times\dot{\mathbf{m}} + \mathbf{m}\times(\mathbf{m}\times(\mathbf{u}\cdot\nabla)\mathbf{m}) + \beta\mathbf{m}\times(\mathbf{u}\cdot\nabla)\mathbf{m}, \tag{50}$$

$$\mathbf{h} = \mathbf{H}_{\text{eff}} + H_{\text{DL}}\mathbf{m}\times\mathbf{p} + H_{\text{FL}}\mathbf{p}. \tag{51}$$

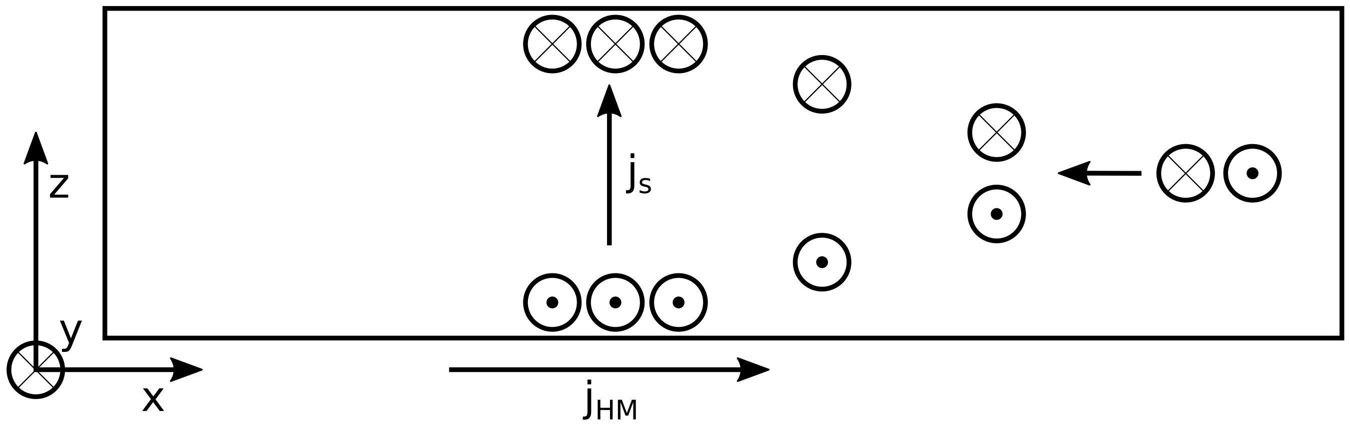


**Figure S11 | Schematic of the spin current generated by spin orbit torques (SOT).** An electrical current $\mathbf{j}_{\text{HM}}$ flows in a heavy metal (HM). Electrons, being negatively charged, move with a velocity antiparallel to $\mathbf{j}_{\text{HM}}$. Here, electrons are depicted as circles and their magnetic moment, pointing in $y$ direction, is indicated with a cross (positive) or dot (negative). In a material with negative spin Hall angle $\theta_{\text{SH}}^{\text{eff}}$, spins with magnetic moments pointing in positive $y$ direction accumulate on the top surface and their counterparts accumulate on the bottom surface. This can be described by a spin current $\mathbf{j}_s$, flowing in $z$ direction with a polarization (of the magnetic moments) in positive $y$ direction.

Here, $\gamma = 1.76 \times 10^{11}$ As/kg is the gyromagnetic ratio and $\mathbf{H}_{\text{eff}}$ is the effective magnetostatic field that includes the effect of anisotropy, exchange, demagnetization, and DMI. $\mathbf{p}$ is a unit vector in the direction of the magnetic moments that are injected into the ferromagnet, determined by $\mathbf{p} = \text{sign}(\theta_{\text{SH}}^{\text{eff}})\hat{\mathbf{j}} \times \mathbf{n}$, where $\mathbf{n}$ is the surface normal of the heavy metal and $\theta_{\text{SH}}^{\text{eff}}$ is the effective spin Hall angle that takes into account the film thickness and the spin mixing conductance at the heavy metal – ferromagnet interface [42]. Assuming a heavy metal below the ferromagnet ($\mathbf{n} = \hat{z}$) and a SOT current pulse in $+x$ direction, we get $\mathbf{p} = -\,\text{sign}(\theta_{\text{SH}}^{\text{eff}})\hat{y}$. The parameters $H_{\text{DL}}$ and $H_{\text{FL}}$ are the field strengths of the damping-like and field-like torques, respectively, and both are proportional to the injected current density. The $H_{\text{DL}}$ is determined via

$$H_{\text{DL}} = \left| \frac{\hbar \theta_{\text{SH}}^{\text{eff}} j_{\text{HM}}}{2\mu_0 e M_s d} \right|, \tag{52}$$

where $\hbar$ is the reduced Planck constant, $j_{\text{HM}}$ is the current density in the heavy metal, $e$ is the elementary charge, $M_s$ is the saturation magnetization, and $d$ is the thickness of a single ferromagnetic layer. Note that the sign of the current and the sign of the spin Hall angle are already taken into account in the polarization vector $\mathbf{p}$, hence the absolute value is taken for $H_{\text{DL}}$. The spin drift velocity in the magnetic layer $\mathbf{u}$ is determined by $\mathbf{u} = -\mathbf{j}_{\text{FM}} \frac{\mu_B P}{|e| M_s (1+\beta^2)}$ with the current density in the magnetic layer $\mathbf{j}_{\text{FM}}$ and its spin polarization of the magnetic layer $P$. Finally, $\beta$ is a phenomenological non-adiabaticity parameter [38, 43, 44].

The derivation of the Thiele equation is explained in a step-by-step analysis in [43]. Here, we briefly recap

the idea. First, we assume a rigid skyrmion moving with fixed velocity $\mathbf{v}$, that is,

$$\dot{\mathbf{m}} = -(\mathbf{v} \cdot \nabla)\mathbf{m}. \tag{53}$$

Then, we can write the LLG equation as

$$-\gamma\mu_0 \mathbf{m} \times \mathbf{h}_{\text{tot}} = 0 \tag{54}$$

with

$$-\gamma\mu_0 \mathbf{h}_{\text{tot}} = -\mathbf{m} \times [(\mathbf{v} - \mathbf{u}) \cdot \nabla]\, \mathbf{m} - [(\alpha\mathbf{v} - \beta\mathbf{u}) \cdot \nabla]\, \mathbf{m} - \gamma\mu_0 \mathbf{h} \tag{55}$$

from which we conclude $\partial_i \mathbf{m} \cdot (-\gamma\mu_0)\mathbf{h}_{\text{tot}} = 0$ for any $i = x, y, z$. By integrating this identity over $x$ and $y$ we obtain the well-known Thiele equation [45]

$$\mathbf{G} \times (\mathbf{v} - \mathbf{u}) - \tilde{D}(\alpha\mathbf{v} - \beta\mathbf{u}) + \mathbf{F} = 0, \tag{56}$$

where each term of the sum originates from the terms in Eq. (55) in the same sequence. The coefficients are the gyrocoupling vector $\mathbf{G} = (0, 0, G)$ with

$$G = -\int \mathrm{d}x\mathrm{d}y\, \mathbf{m} \cdot (\partial_x \mathbf{m} \times \partial_y \mathbf{m}) = -4\pi N \tag{57}$$

with the topological charge $N$ (equal to the polarity for defect-free skyrmions) and the dissipation integral [44]

$$D = \int \mathrm{d}x\mathrm{d}y\, (\partial_x \mathbf{m})^2 = \frac{1}{2}\int \mathrm{d}x\mathrm{d}y\, \left[(\partial_x \mathbf{m})^2 + (\partial_y \mathbf{m})^2\right] = \pi I_A(\rho) \tag{58}$$

with the exchange integral $I_A(\rho)$ (where the factor $2\pi$ comes from switching to polar coordinates). The

interesting factor is the accelerating force [46], which we first express for a current in $+x$ direction:

$$F_i(\hat{\boldsymbol{j}}_{\text{HM}} = +\hat{x}) = \int \mathrm{d}x\mathrm{d}y\, (\partial_i \mathbf{m}) \cdot (-\gamma\mu_0)\mathbf{h} \tag{59}$$

$$= -\gamma\mu_0 H_{\text{DL}} \int \mathrm{d}x\mathrm{d}y\, (\partial_i \mathbf{m}) \cdot (\mathbf{m} \times \mathbf{p}) \tag{60}$$

$$= -\gamma\mu_0 H_{\text{DL}}\, \text{sign}(\theta_{\text{SH}}^{\text{eff}}) \int \mathrm{d}x\mathrm{d}y\, (m_z \partial_i m_x - m_x \partial_i m_z) \tag{61}$$

$$\mathbf{F}(\hat{\boldsymbol{j}}_{\text{HM}} = +\hat{x}) = -\gamma\mu_0 H_{\text{DL}}\, \text{sign}(\theta_{\text{SH}}^{\text{eff}}) \int \mathrm{d}x\mathrm{d}y\, (m_z \nabla m_x - m_x \nabla m_z) \tag{62}$$

where we used that the field-like term $\mathbf{p}$ is translationally invariant (i.e., can be moved out of the integral) and $\int(\partial_i \mathbf{m}) = 0$ by partial integration and constant $\mathbf{m}$ along the boundaries. For $\mathbf{H}_{\text{eff}}$ we assumed the same translational invariance (that is, field gradients are not included at this stage). The integral can be written in polar coordinates with $m_x = \sin\theta\cos(\varphi + \psi)$ and $m_z = \cos\theta$

$$\int \mathrm{d}x\mathrm{d}y\; [m_z \nabla m_x - m_x \nabla m_z] = \int r\mathrm{d}r\mathrm{d}\varphi \left[m_z \left(\hat{\mathbf{r}}\partial_r + \hat{\boldsymbol{\varphi}}\frac{1}{r}\partial_\varphi\right) m_x - m_x \left(\hat{\mathbf{r}}\partial_r + \hat{\boldsymbol{\varphi}}\frac{1}{r}\partial_\varphi\right) m_z\right] \tag{63}$$

$$= \int \mathrm{d}r\mathrm{d}\varphi \left[\hat{\mathbf{r}}\cos(\varphi + \psi) r\partial_r\theta - \hat{\boldsymbol{\varphi}}\cos\theta\sin\theta\sin(\varphi + \psi)\right]. \tag{64}$$

The integral with respect to $\varphi$ has to take into consideration that $\hat{\mathbf{r}}$ and $\hat{\boldsymbol{\varphi}}$ depend on $\varphi$. Specifically, we first note that $\hat{\boldsymbol{\varphi}} = \partial_\varphi \hat{\mathbf{r}}$ and hence

$$\int \mathrm{d}\varphi\, \hat{\boldsymbol{\varphi}} \sin(\varphi + \psi) = -\int \mathrm{d}\varphi\, \hat{\mathbf{r}}\cos(\varphi + \psi) \tag{65}$$

obtained by integration by parts. Furthermore,

$$\int \mathrm{d}\varphi\, \hat{\mathbf{r}}\cos(\varphi + \psi) = \tilde{R}(\psi) \int \mathrm{d}\varphi\, \hat{\mathbf{r}}\cos\varphi \tag{66}$$

$$= \tilde{R}(\psi) \int \mathrm{d}\varphi\; (\cos\varphi\hat{\mathbf{x}} + \sin\varphi\hat{\mathbf{y}})\cos\varphi \tag{67}$$

$$= \pi\tilde{R}(\psi)\hat{\mathbf{x}} = \pi\tilde{R}(\psi)\hat{\mathbf{j}}_{\text{HM}}, \tag{68}$$

where the last equation is true for any direction of $\hat{\boldsymbol{j}}_{\mathrm{HM}}$ and where

$$\tilde{R}(\psi) = \begin{pmatrix} \cos\psi & \sin\psi \\ -\sin\psi & \cos\psi \end{pmatrix} \tag{69}$$

is the rotation matrix corresponding to the domain wall angle. With this, the integral in Eq. (64) simplifies to

$$\pi\tilde{R}(\psi)\hat{\mathbf{j}}_{\mathrm{HM}} \int \mathrm{d}r\,[r\partial_r\theta - \cos\theta\sin\theta] = \pi\tilde{R}(\psi)\hat{\mathbf{j}}_{\mathrm{HM}}\Delta N I_D(\rho), \tag{70}$$

where we have identified the integral as being the same as the DMI integral and added a factor $N$ (skyrmion topological charge) to also cover the case of negative skyrmion polarity, i.e., $m_z$ pointing down in the center. Putting it all together, we thus have

$$\mathbf{F} = -\frac{\hbar\gamma\pi N\theta_{\mathrm{SH}}^{\mathrm{eff}} N}{2|e|M_s d}\Delta I_D(\rho)\tilde{R}(\psi)\mathbf{j}_{\mathrm{HM}}. \tag{71}$$

The direction of the force $\mathbf{F}$ can be easily determined by noting that $\tilde{R}$ rotates the current vector $\mathbf{j}_{\mathrm{HM}}$ by $-\psi$, or clockwise by $\psi$, and that a negative $\theta_{\mathrm{SH}}^{\mathrm{eff}}$ and a negative $N$ can both flip the direction of $\mathbf{F}$. For instance, a skyrmion with core magnetization pointing down ($N < 0$) with $\psi = 0$ feels a force in the direction of $\mathbf{j}_{\mathrm{HM}}$ if the spin Hall angle is positive, and so does skyrmion with positive polarity and $\psi = \pi$. Both of these skyrmions are called left-handed. Their motion with the current for Pt (positive spin Hall angle) and against the current for Ta (negative spin Hall angle) is in agreement with experimental observations [14, 47, 48].

The Thiele equation predicts a finite angle $\xi$ between the applied force and the skyrmion velocity $\mathbf{v}$, which is called skyrmion Hall angle. One can easily show[1] that Eq. (56) leads to

$$\xi = \mathrm{atan2}(\alpha, g), \tag{72}$$

$$g(\rho) = \frac{G}{\tilde{D}} = -\frac{4N}{I_A(\rho)}. \tag{73}$$

With the large $R$ approximation for $I_A$, this reduces to the well-known formula [49]

$$\tan(\xi) \approx -\frac{2\Delta N}{\alpha R} \tag{74}$$

[1]Set $\mathbf{F} = F\hat{x}$ and obtain $\tan(\xi) = v_x/v_y$ from the $y$ component of the Thiele equation.

for bubble skyrmions. Note that the sign in Eq. 73 means that skyrmions with a core magnetization pointing up ($N = 1$) are deflected to negative $y$ when moving in $+x$ direction and skyrmions with a core magnetization pointing down ($N = -1$) are deflected to positive $y$ when moving in $+x$ direction, which is consistent with the observations of Litzius *et al.* and Jiang *et al.* [47, 48]. Finally, combining all the analysis above leads to the steady state velocities $v_x$ and $v_y$ for a skyrmion of arbitrary domain wall angle $\psi$ driven by a current in $x$ direction $\mathbf{j}_{\mathrm{HM}} = j_{\mathrm{HM}}\hat{x}$ with a (positive) mobility $\mu$:

$$\mathbf{v} = v(\cos\xi', \sin\xi'), \tag{75}$$

$$v = \mu j_{\mathrm{HM}}, \tag{76}$$

$$\mu = \frac{1}{\sqrt{g(\rho)^2 + \alpha^2}} \frac{\gamma\hbar N|\theta_{\mathrm{SH}}^{\mathrm{eff}}|\Delta I_D(\rho)}{2|e|dM_s I_A(\rho)}. \tag{77}$$

Here, $\xi'$ is the angle between the skyrmion velocity and the applied current direction,

$$\xi' = \xi - \psi + \pi\Theta(\theta_{\mathrm{SH}}^{\mathrm{eff}}N), \tag{78}$$

where $\Theta$ is the Heaviside step function. In most experiments, $\xi'$ (and not $\xi$) is the observable quantity. It depends on the domain wall angle $\psi$ as well as on radius and domain wall width. Essentially, $-\psi + \pi\Theta(\theta_{\mathrm{SH}}^{\mathrm{eff}}N)$ is the angle of the force $\mathbf{F}$ with respect to the direction of current. We check that the sign is correct using the example of a left-handed skyrmion in Pt/CoFeB/MgO that should move with the current. Indeed, for $N = 1$, $\psi = \pi$ and $\theta_{\mathrm{SH}}^{\mathrm{eff}} > 0$, we obtain $-\psi + \pi\Theta(\theta_{\mathrm{SH}}^{\mathrm{eff}}N) = 0$, i.e., the force points in the direction of current as expected.

Finally, we discuss how to treat multilayers with non-uniform magnetization along $z$, in particular synthetic antiferromagnets (SAF). We illustrate the concept on a 2-layer SAF and for $\mathbf{u} = 0$. Consider a SAF structure with two magnetic layers with thickness $d_1$ and $d_2$ and saturation magnetization $M_{s1}$ and $M_{s2}$, where the interlayer exchange coupling is strong enough to ensure the same (but inverted) spin structure in both layers. According to Newton's law, the sum of all *forces* should be zero. Now we need to keep in mind that Eq. (56) was normalized by the common factor $dM_s/\gamma$ in all terms (this is why Eq. (56) has units of velocity and not force). When adding forces of layers with different $d$ and $M_s$, this factor needs to be considered. The result

reads

$$\frac{d_1 M_{s1}}{\gamma}\left(\mathbf{G}_1 \times \mathbf{v} - \tilde{D}_1 \alpha \mathbf{v} + \mathbf{F}_1\right) + \frac{d_2 M_{s2}}{\gamma}\left(\mathbf{G}_2 \times \mathbf{v} - \tilde{D}_2 \alpha \mathbf{v} + \mathbf{F}_2\right) = 0. \tag{79}$$

Introducing $r := \frac{d_1 M_{s1}}{d_2 M_{s2}}$ and realizing that $\mathbf{G}_1 = -\mathbf{G}_2 = \mathbf{g}\tilde{D}$, $\tilde{D}_1 = \tilde{D}_2 = \tilde{D}$, and $\mathbf{F}_1 = r\mathbf{F}_2 = \mathbf{f}_1\tilde{D}$, this simplifies to

$$\frac{1-r}{1+r}\mathbf{g} \times \mathbf{v} - \alpha \mathbf{v} + \frac{2}{1+r}\mathbf{f}_1 = 0. \tag{80}$$

That is, we can use the same results as obtained before by replacing $\mathbf{g}$ by $\frac{1-r}{1+r}\mathbf{g}$ and $\mathbf{f}$ by $\frac{2}{1+r}\mathbf{f}_1$. The index "1" at $\mathbf{f}_1$ reminds us that $d_1$ and $M_{s1}$ should be used to calculate this term. Very similarly, one obtains the general result for $\mathcal{N}$ as presented in the main text.

# S11 Detailed steps to solve the energy integrals

## S11.1 Exchange energy

The exchange energy is given in cylindrical coordinates by

$$\begin{aligned}
E_A &= A \int r \mathrm{d}r \mathrm{d}z \mathrm{d}\varphi \left[(\partial_r \theta)^2 + r^{-2} \sin^2 \theta\right] && (81)\\
&= 2\pi A d \int_0^\infty \mathrm{d}r \left[r(\partial_r \theta)^2 + r^{-1} \sin^2 \theta\right] && (82)\\
&= 2\pi A d \int_0^\infty \mathrm{d}\varrho \left[\varrho(\partial_\varrho \theta)^2 + \varrho^{-1} \sin^2 \theta\right] && (83)\\
&= 2\pi A d I_A, && (84)
\end{aligned}$$

where we carried out the trivial $\varphi$ and $z$ integrations in the first step. In the second step we substituted the dimensional variable $r$ by the dimensionless quantity $\varrho = r/\Delta$. There is no obvious analytical expression for $I_A$, but numerical evaluation suggests that good approximation is given by

$$I_A \approx 2\rho + 2/\rho + 1.93(\rho - 0.65)\exp(-1.48(\rho - 0.65)). \tag{85}$$

## S11.2 Anisotropy energy

The anisotropy energy integral can be expressed analytically:

$$E_K = 2\pi d K_u \int_0^\infty \mathrm{d}r r \sin^2\theta \tag{86}$$

$$= 2\pi K_u d \Delta^2 \int_0^\infty \mathrm{d}\varrho \varrho \sin^2\theta \tag{87}$$

$$= 2\pi K_u d \Delta^2 I_K, \tag{88}$$

$$I_K = \frac{4e^{3\rho_0}\cosh(\rho_0)\left(-\mathrm{Li}_2\left(-e^{-2\rho_0}\right) + \mathrm{Li}_2\left(-e^{2\rho_0}\right) + 2\left(\ln\left(e^{2\rho_0}+1\right) - \rho_0\right)\sinh(2\rho_0)\right)}{\left(e^{2\rho_0}-1\right)^3} \tag{89}$$

However, the expression is complicated and difficult to evaluate numerically for large $\rho$ because it involves differences of almost identical numbers. Therefore, we suggest to use a simplified version instead:

$$I_K \approx 2\rho - \frac{1}{3}\exp\left(-\rho/\sqrt{2}\right). \tag{90}$$

## S11.3 DMI

The general form of a Dzyaloshinskii-Moriya interaction energy density can be expressed as a linear combination of the Lifshitz invariants $\mathcal{L}_{ij}^{(k)} = m_i \partial_k m_j - m_j \partial_k m_i$, with $i, j, k \in \{x, y, z\}$ and $i > j$ [50, 51]. If we assume no gradient of $\mathbf{m}$ along the $z$ direction and isotropic interaction in the $xy$ plane (i.e., interactions that allow for the formation of circular skyrmions), the two terms that remain are the ones that are symmetric under exchange of $x$ and $y$, namely $\mathcal{L}_i = \mathcal{L}_{zx}^x + \mathcal{L}_{zy}^y$ and $\mathcal{L}_b = \mathcal{L}_{zy}^x + \mathcal{L}_{zx}^y$ [5, 50]. The total DMI energy density is given by the linear combination $w_{\mathrm{DMI}} = D_i \mathcal{L}_i + D_b \mathcal{L}_b$. Both interaction energies are minimized by a constant domain wall angle $\psi$. $\mathcal{L}_i$ favors Néel skyrmions and $\mathcal{L}_b$ favors Bloch skyrmions. A non-zero $D_i$ is mostly found in multilayer systems where the DMI originates from the interface whereas a non-zero $D_b$ is characteristic of bulk DMI due to a $\mathrm{D_n}$ symmetry of the crystal [5]. In cylindrical coordinates, the total DMI energy reads [5]

$$E_D = -2\pi d \Delta (D_i \cos\psi - D_b \sin\psi) \int_0^\infty \mathrm{d}\varrho \left[\varrho\, \partial_\varrho \theta + \sin\theta\cos\theta\right] \tag{91}$$

$$= -2\pi d \Delta (D_i \cos\psi - D_b \sin\psi) I_D. \tag{92}$$

The minus sign is convention. It leads to right-handed domain walls at positive $D_i$ ($\psi = 0$ when the skyrmion core points upward) and clockwise ($\psi = 3\pi/2$) skyrmions for positive $D_b$. The integral $I_D$ has an analytical solution:

$$I_D = \frac{\cosh\rho_0}{\sinh^2\rho_0}\left(\frac{4\arctan\left(e^{\rho_0}\right) - \pi}{\sinh\rho_0} - 2\right) - \frac{e^{3\rho_0}}{2}\Phi\left(-e^{2\rho_0}, 2, \frac{3}{2}\right) \qquad (93)$$
$$+ i\mathrm{Li}_2\left(-ie^{-\rho_0}\right) - i\mathrm{Li}_2\left(ie^{-\rho_0}\right) + 2e^{\rho_0},$$

where $\Phi(z, s, a) = \sum_{k=0}^{\infty} \frac{z^k}{(a+k)^s}$ is the Lerch transcendent function, but again we can approximate the result with less than 1 % error for all $\rho$ by a much simpler function:

$$I_D \approx \pi\rho + \frac{1}{2}\exp\left(-\rho\right). \qquad (94)$$

## S11.4 Zeeman energy

The Zeeman energy density is given by the product of the local magnetization and the applied magnetic field $B_z$, assuming that the field is applied in $z$ direction and that a positive $B_z$ indicates that the field is aligned with the magnetization in the core of the skyrmion (thus favoring larger skyrmions). By subtracting the reference energy of a homogeneously magnetized film, we obtain the Zeeman energy associated with the presence of the skyrmion:

$$E_Z = -2\pi M_s B_z d\Delta^2 \int_0^\infty \mathrm{d}\varrho\varrho\left[1 - m_z\left(\varrho, \rho_0\right)\right] \qquad (95)$$
$$= -2\pi M_s B_z d\Delta^2 I_Z, \qquad (96)$$
$$I_Z = \frac{1}{2}\left[\mathrm{Li}_2\left(-e^{-2\rho_0}\right) - \mathrm{Li}_2\left(-e^{2\rho_0}\right)\right]\coth(\rho_0) \qquad (97)$$
$$\approx \rho^2 + \frac{\pi^2}{12} - 0.21\exp(-\rho^2) \qquad (98)$$

The last line is again an accurate approximation of the otherwise difficult to evaluate exact expression for $I_Z$.

### 11.4.1 Surface stray fields

As derived by Tu [6], the surface stray field energy of any circular symmetric structure can be calculated in cylindrical coordinates via

$$E_{d,s} = -2\pi\mu_0 M_s^2 d\Delta^2 I_s \tag{99}$$

$$I_s = \frac{1}{2}\int_0^\infty \mathrm{d}\varrho\varrho\left[1 - m_z\left(\varrho,\rho_0\right) + m_z\left(\varrho,\rho_0\right) f(\varrho,t)\right] \tag{100}$$

$$f(\varrho,t) = t^{-2}\int_0^\infty \mathrm{d}x\, x\, g\left(\tfrac{x}{t},\tfrac{\varrho}{t}\right)\left[1 - m_z(x,\rho_0)\right]. \tag{101}$$

$$g(y,z) = \int_0^\infty J_0(xy)J_0(xz)(1-e^{-x})\mathrm{d}x \tag{102}$$

where $t = d/\Delta$ and $J_0$ is the Bessel function of the first kind of zeroth order. Note that here, the energy integrals of a skyrmion are expressed by normalizing all length units to the domain wall width $\Delta$, which is in contrast to Tu's original work [6] where length units are normalized to the sample thickness $d$. This leads to the factor $t^{-2}$ in the function $f(\varrho,t)$.

The integral over the Bessel functions can be done analytically, see [52] p. 249:

$$g(y,z) = \frac{1}{\pi\sqrt{yz}}\left[k_0\left(y,z\right)K\left(k_0\left(y,z\right)\right) - k_1\left(y,z\right)K\left(k_1\left(y,z\right)\right)\right], \tag{103}$$

$$k_p(y,z) = \sqrt{\frac{4yz}{p^2 + (y+z)^2}}. \tag{104}$$

The goal is to calculate $E_d$ with a relative error of less than 0.1 %. Solving the double integral numerically requires some tricks. First, we note that the integral over $g$ alone is normalized to 1 and that $|1 - m_z(x+\rho_0,\rho_0)| < 10^{-12}$ for all $x > 15$ and decaying exponentially with $x$, which means that we can replace the upper integral limit in (101) by $\rho_0 + 15$ with negligible error compared to our precision goal of $10^{-3}$. Second, for large $\rho_0$, $m_z = \cos(\theta)$ with $\theta$ from Eq. (3) of the main text contains differences of exponentials with large arguments, which is difficult to evaluate numerically. Instead, we write $m_z$ as

$$m_z(\varrho,\rho_0) = \frac{4\sinh^2(\varrho)}{\cosh(2\rho_0) + \cosh(2\varrho)} - 1 \tag{105}$$

which can be approximated for $\rho_0 \geq 5$ by the Fermi function

$$m_{z,s}(\varrho, \rho_0) = \frac{2}{1 + \exp(2(\rho_0 - \varrho))} - 1 \tag{106}$$

which can be easily evaluated for all $\varrho$ and $\rho_0$. Third, the function $g\left(\frac{x}{t}, \frac{\varrho}{t}\right)$ has a singularity at $x = \varrho$ that is difficult to evaluate in a numerical integration. However, in a small interval $x \in [\max(0, \varrho - \epsilon), \varrho + \epsilon]$ around the divergence, $m_z$ changes at most by the maximum derivative $\max(\partial_x m_z(x, \rho_0))$ times the interval length $2\epsilon$. Hence, we can approximate $1 - m_z$ to be constant with a relative error of less than $\left(2\epsilon \max\left|\frac{\partial_\varrho m_z(\varrho,\rho_0)}{1-m_z(\varrho,\rho_0)}\right|\right) < 4\epsilon$. In our calculations, we choose $\epsilon = 10^{-7}$ and the error made by approximating $m_z$ to be constant is negligible. Hence, we can write

$$t^{-2}\int_{\max(0,\varrho-\epsilon)}^{\varrho+\epsilon} \mathrm{d}x\, x\, g\left(\tfrac{x}{t}, \tfrac{\varrho}{t}\right)[1 - m_z(x, \rho_0)] \approx [1 - m_z(\varrho, \rho_0)]t^{-2}\int_{\varrho-\epsilon}^{\varrho+\epsilon} \mathrm{d}x\, x\, g\left(\tfrac{x}{t}, \tfrac{\varrho}{t}\right) \tag{107}$$

$$= [1 - m_z(\varrho, \rho_0)]G(\varrho, t) \tag{108}$$

with

$$G(\varrho, t) = t^{-2}\int_{\max(0,\varrho-\epsilon)}^{\varrho+\epsilon} \mathrm{d}x\, x\, g\left(\tfrac{x}{t}, \tfrac{\varrho}{t}\right) \tag{109}$$

$$= \int_{\max(0,\varrho/t-\epsilon/t)}^{\varrho/t+\epsilon/t} \mathrm{d}x\, x\, g\left(x, \tfrac{\varrho}{t}\right) \tag{110}$$

$$= \int_0^a \mathrm{d}x\, x\, g\left(x, \tfrac{\varrho}{t}\right) - \int_0^{\max(0,\varrho/t-\epsilon/t)} \mathrm{d}x\, x\, g\left(x, \tfrac{\varrho}{t}\right) - \int_{\varrho/t+\epsilon/t}^a \mathrm{d}x\, x\, g\left(x, \tfrac{\varrho}{t}\right). \tag{111}$$

The first term in Eq. (111) has an analytic solution (assuming $a > \varrho/t$):

$$\int_0^a \mathrm{d}x\, x\, g\left(x, \tfrac{\varrho}{t}\right) = \sum_{m=0}^{\infty} c_m, \tag{112}$$

$$c_m = a\, C_m^2 \frac{(m+1)^2}{1-2m}\left(-\frac{\varrho}{4at}\right)^{2m} \tag{113}$$

$$- a^2 C_m \frac{m+1}{2}\, {}_2F_1(m + \tfrac{1}{2}, m+1, 2, -a^2)\left(-\frac{\varrho}{4t}\right)^m, \tag{114}$$

where $C_m$ is the Catalan number and ${}_2F_1$ is the Gauss hypergeometric function. For $a = 10\varrho/t + 1$, the sum converges to 10 digits precision after three terms. Hence, we set $a = 10\varrho/t + 1$ and stop the sum after the $m = 3$ term. Furthermore, we write the $m = 0$ term explicitly in a form that can be evaluated accurately with

64 bit floating point numbers:

$$c_0 = \begin{cases} 1 + \varrho/t - \sqrt{1 + \varrho/t} & \varrho/t < 10^{15} \\ 1 & \text{else.} \end{cases} \tag{115}$$

The remaining two terms in Eq. (111) are integrals of a finite valued function over a finite interval and are evaluated numerically. Finally, we split the integral $I_s$ in two parts

$$I_s = \frac{1}{2}\int_0^\infty \mathrm{d}\varrho\varrho\left[1 - m_z(\varrho,\rho_0) + m_z(\varrho,\rho_0)\, f(\varrho,t)\right] = I_{s1} + I_{s2} \tag{116}$$

with

$$I_{s1}(\rho_0,t) = \frac{1}{2}\int_0^{10\rho_0+150} \mathrm{d}\varrho\varrho\left[1 - m_z(\varrho,\rho_0) + m_z(\varrho,\rho_0)\, f(\varrho,t)\right], \tag{117}$$

$$I_{s2}(\rho_0,t) = \frac{1}{2}\int_{10\rho_0+150}^\infty \mathrm{d}\varrho\varrho\, f_a(\varrho,t), \tag{118}$$

where we have used that $m_z(\varrho,\rho_0) \approx 1$ in the limits of the integration of $I_{s2}$. The function $f_a(\varrho,t)$ is an analytic approximation of $f(\varrho,t)$, which is based on a Taylor expansion of $g\left(\frac{x}{t},\frac{\varrho}{t}\right)$ for small $x$. In particular, for $x < 0.1\varrho$, a second order polynomial approximates $g\left(\frac{x}{t},\frac{\varrho}{t}\right)$ with less than $10^{-4}$ relative error. The polynomial approximation is

$$g(x,\varrho) \approx g_a(x,\varrho) = g_{a0}(\varrho) + x^2 g_{a2}(\varrho), \tag{119}$$

$$g_{a0}(\varrho) = \frac{1}{\varrho} - \sqrt{\frac{1}{\varrho^2+1}} \tag{120}$$

$$g_{a2}(\varrho) = \frac{1}{4}\left(\frac{1}{\varrho^3} + \frac{2-\varrho^2}{\left(\varrho^2+1\right)^{5/2}}\right) \tag{121}$$

and the integral

$$f_a(\varrho, \rho_0, t) = t^{-2} \int_0^{\rho_0+15} \mathrm{d}x x g_a\left(\tfrac{x}{t}, \tfrac{\varrho}{t}\right)[1 - m_z(x, \rho_0)] \tag{122}$$

$$= \frac{1}{16t}\coth(\rho_0)\left[3\left(\frac{1}{\varrho^3} + \frac{2t^2 - \varrho^2}{(\varrho^2 + t^2)^{5/2}}\right)\left(\mathrm{Li}_4\left(-e^{-2\rho_0}\right) - \mathrm{Li}_4\left(-e^{2\rho_0}\right)\right)\right. \tag{123}$$

$$\left. - 8\left(\frac{1}{\sqrt{\varrho^2 + t^2}} - \frac{1}{\varrho}\right)\left(\mathrm{Li}_2\left(-e^{-2\rho_0}\right) - \mathrm{Li}_2\left(-e^{2\rho_0}\right)\right)\right]. \tag{124}$$

Note, in particular, that for very large $t$, the second order term $g_{a2}$ becomes insignificant because it scales with $t^{-2}$. The zeroth order term does not depend on $x$, and the integral $f_a$, after neglecting the second order term, separates into two factors

$$f_a(\varrho, \rho_0, t \to \infty) = t^{-2} g_{a0}(\varrho/t) \int_0^{\infty} \mathrm{d}x x[1 - m_z(x, \rho_0)]. \tag{125}$$

The integral in Eq. (125) does not depend on $\varrho$ and the integral over the first factor in Eq. (125) becomes one in the limit $t \to \infty$, independent of the lower limit of the integration. Hence,

$$I_{s2}(\rho_0, t \to \infty) = \int_0^{\infty} \mathrm{d}x x[1 - m_z(x, \rho_0)] \int_{10\rho_0+150}^{\infty} \mathrm{d}\varrho \varrho t^{-2} g_{a0}(\varrho/t) \tag{126}$$

$$\to \int_0^{\infty} \mathrm{d}x x[1 - m_z(x, \rho_0)] \tag{127}$$

which is exactly the same as $I_{s1}$ in the limit $t \to \infty$, when $f \to 0$. Hence,

$$\lim_{t\to\infty} I_s(\rho_0, t) = \int_0^{\infty} \mathrm{d}x x[1 - m_z(x, \rho_0)] \tag{128}$$

$$= \frac{1}{2}\left[\mathrm{Li}_2\left(-e^{-2\rho_0}\right) - \mathrm{Li}_2\left(-e^{2\rho_0}\right)\right]\coth(\rho_0). \tag{129}$$

### 11.4.2 Volume stray fields

As for the surface stray field energy, we aim to derive the volume stray field energy with a precision of $10^{-3}$. In general, the stray field energy of volume charges can be calculated via [43]:

$$E_d = \frac{\mu_0}{8\pi} \int \mathrm{d}^3\mathbf{r}\mathrm{d}^3\mathbf{r}'(\mathrm{div}\,\mathbf{M}(\mathbf{r}))(\mathrm{div}\,\mathbf{M}(\mathbf{r}'))\frac{1}{|\mathbf{r} - \mathbf{r}'|}. \tag{130}$$

In cylindrical coordinates, and using that $\mathbf{M}(\mathbf{r}) = \mathbf{M}(r)$, this simplifies to

$$E_d = \frac{\mu_0}{8\pi} \int_0^\infty \mathrm{d}r \partial_r (r M_r(r)) \int_0^\infty \mathrm{d}r' \partial_{r'} (r' M_r(r')) \tag{131}$$

$$\times \int_0^{2\pi} \mathrm{d}\phi \int_0^{2\pi} \mathrm{d}\phi' \int_{-d/2}^{d/2} \mathrm{d}z \int_{-d/2}^{d/2} \mathrm{d}z' \frac{1}{\sqrt{r^2 + r'^2 - 2rr' \cos(\phi - \phi') + (z - z')^2}} \tag{132}$$

$$= \frac{\mu_0 \Delta^3}{8\pi} \int_0^\infty \mathrm{d}\varrho \partial_\varrho (\varrho M_r(\varrho)) \int_0^\infty \mathrm{d}\varrho' \partial_{\varrho'} (\varrho' M_r(\varrho')) \tag{133}$$

$$\times \int_0^{2\pi} \mathrm{d}\phi \int_0^{2\pi} \mathrm{d}\phi' \int_{-\frac{t}{2}}^{\frac{t}{2}} \mathrm{d}z \int_{-\frac{t}{2}}^{\frac{t}{2}} \mathrm{d}z' \frac{1}{\sqrt{\varrho^2 + \varrho'^2 - 2\varrho\varrho' \cos(\phi - \phi') + (z - z')^2}} \tag{134}$$

The integration with respect to $z$ and $z'$ and $\phi'$ can be carried out analytically, yielding the integration kernel $k(\varrho, \varrho', \phi, t)$:

$$k(\varrho, \varrho', \phi, t) = \frac{1}{2\pi} \int_0^{2\pi} \mathrm{d}\phi' \int_{-\frac{t}{2}}^{\frac{t}{2}} \mathrm{d}z \int_{-\frac{t}{2}}^{\frac{t}{2}} \mathrm{d}z' \frac{1}{\sqrt{\varrho^2 + \varrho'^2 - 2\varrho\varrho' \cos(\phi - \phi') + (z - z')^2}} \tag{135}$$

$$= -2\sqrt{t^2 + \varrho^2 - 2\varrho\varrho' \cos(\phi) + \varrho'^2} \tag{136}$$

$$+ 2\sqrt{\varrho^2 - 2\varrho\varrho' \cos(\phi) + \varrho'^2} \tag{137}$$

$$- \frac{t}{2} \Big[ \ln\left(\sqrt{t^2 + \varrho^2 - 2\varrho\varrho' \cos(\phi) + \varrho'^2} - t\right) \tag{138}$$

$$- 3\ln\left(\sqrt{t^2 + \varrho^2 - 2\varrho\varrho' \cos(\phi) + \varrho'^2} + t\right) \tag{139}$$

$$+ \ln\left(\varrho^2 - 2\varrho\varrho' \cos(\phi) + \varrho'^2\right) \Big] \tag{140}$$

Together with expression for the in-plane spin orientation $m_{\mathrm{ip}}$

$$M_r(\varrho) = M_s \cos(\psi) m_{\mathrm{ip}}(\varrho) \tag{141}$$

$$m_{\mathrm{ip}}(\varrho) = \sin(\theta(\varrho)) \tag{142}$$

$$= -2 \frac{\cosh(\rho_0) \sinh(\varrho)}{\cosh(\varrho - \rho_0) \cosh(\rho_0 + \varrho)} \tag{143}$$

and hence

$$
\begin{aligned}
g_v(\varrho, \rho_0) :&= \partial_\varrho(\varrho m_{\mathrm{ip}}(\varrho)) && (144)\\
&= -2\frac{\cosh(\rho_0)}{\cosh(\varrho-\rho_0)\cosh(\rho_0+\varrho)} && (145)\\
&\quad\times(\sinh(\varrho)+\varrho\,[\cosh(\varrho)-\sinh(\varrho)(\tanh(\varrho-\rho_0)+\tanh(\rho_0+\varrho))]) && (146)
\end{aligned}
$$

this leads to a lengthy, but numerically solvable threefold integral for the volume stray field energy:

$$
\begin{aligned}
E_{d,v} &= 4\pi\mu_0 M_s^2 \Delta R d \cos^2(\psi) I_v, && (147)\\
I_v &= \frac{1}{8\pi\rho d}\int_0^\infty \mathrm{d}\varrho \int_0^\varrho \mathrm{d}\varrho' \int_0^\pi \mathrm{d}\phi\, g_v(\varrho,\rho_0) g_v(\varrho',\rho_0) k(\varrho,\varrho',\phi,t) && (148)
\end{aligned}
$$

Here, we have used that the integrand is periodic in $\phi$ with a periodicity of $\pi$ to write $\int_0^{2\pi}\mathrm{d}\phi$ as $2\int_0^{\pi}\mathrm{d}\phi$. In addition, the integrand is symmetric under exchange of $\varrho$ and $\varrho'$ and therefore the integral $\int_0^\infty \mathrm{d}\varrho \int_0^\infty \mathrm{d}\varrho'$ could be replaced by $\frac{1}{2}\int_0^\infty \mathrm{d}\varrho \int_0^\varrho \mathrm{d}\varrho'$. We have included the factor $\frac{1}{8\pi\rho d}$ in $I_v$ because in this notation, $I_v(\rho\to\infty)$ equals the transverse anisotropy constant $K_\perp$ of a straight domain wall in a single layer film normalized to $\mu_0 M_s^2$:

$$
\lim_{\rho\to\infty} I_v(\rho, t) = \frac{K_\perp}{\mu_0 M_s^2}. \quad (149)
$$

As for the surface stray field energy, a number of considerations are required to make the integral numerically stable for all $\rho_0$ and $t$. First, we note that the maximum value of $g_v(\varrho,\rho_0)$ can be estimated by

$$
\frac{\rho_0}{2} < \max_\varrho |g_v(\varrho,\rho_0)| < \frac{\rho_0}{2} + 1.5 \quad (150)
$$

and that the value of $|g_v(\varrho,\rho_0)|$ at $\varrho = \rho_0 \pm 50$ is negligible compared to the maximum value:

$$
\begin{aligned}
&\frac{|g_v(\rho_0+50,\rho_0)|}{\max_\varrho |g_v(\varrho,\rho_0)|} < 10^{-19} \quad \forall \rho_0 && (151)\\
&\frac{|g_v(\rho_0-50,\rho_0)|}{\max_\varrho |g_v(\varrho,\rho_0)||} < 10^{-19} \quad \forall \rho_0 > 50 && (152)
\end{aligned}
$$

Furthermore, $g_v(\varrho, \rho_0)|$ decays exponentially with increasing $|\varrho - \rho_0|$ for $|\varrho - \rho_0| > 50$. Hence, we can narrow the integration domain of the $\varrho$ integral to $\max[0, \rho_0 - 50] < \varrho < \rho_0 + 50$. Second, for $\rho_0 > 55$, the function for $g_v(\varrho, \rho_0)|$ can be approximated with $< 3 \times 10^{-5}$ relative error by

$$g_{v,a}(\varrho, \rho_0)| = \frac{\varrho \tanh(\varrho - \varrho_0) - 1}{\cosh(\varrho - \varrho_0)} \tag{153}$$

which can be easily evaluated even for very large $\varrho$ and $\rho_0$. Therefore, we use $g_{v,a}(\varrho, \rho_0)|$ instead of $g_v(\varrho, \rho_0)|$ for $\rho_0 > 55$.

With these two approximations, both negligible compared to our precision goal of $10^{-3}$, $I_v$ can be evaluated numerically for $\rho_0 \leq 1000$ and $10^{-4} < t < 10^4$. For $t < 10^{-4}$, $I_v(\rho, t) \propto t$ can be obtained by linear extrapolation. Similarly, for $t > 10^4$, $I_v(\rho, t)$ is constant and can also be obtained by extrapolation. For larger $\rho_0 > 1000$, the curvature of the domain wall becomes insignificant and $I_v$ can be obtained from the a straight wall approximation, i.e., $I_v$ becomes independent of $\rho$. In particular, starting from about $\rho_0 \approx \rho = 100$, the volume stray field energy is in excellent agreement with the volume stray field energy of a straight domain wall, which has a known analytic form [7]:

$$I_{v,\mathrm{DW}}(\tau) = \frac{\ln\left(\frac{\pi}{A^6}\right)}{4\tau} + \ln\Gamma(\tau + 1) - \ln\Gamma\left(\tau + \frac{1}{2}\right) + \frac{7\ln(2)}{24\tau} \tag{154}$$

$$+ \frac{\psi^{(-2)}\left(\tau + \frac{1}{2}\right)}{\tau} - \frac{\psi^{(-2)}(\tau + 1)}{\tau}, \tag{155}$$

where $\tau = \frac{t}{2\pi}$, $A$ is the Glaisher constant, $\Gamma$ the gamma function, and $\psi^{-2}(z) = \int_0^z \mathrm{d}t \ln\Gamma(t)$ the second anti-derivative of the digamma function. Our universal function $I_{v,a}(\rho, t)$ in the main text yields accurate approximations for all $\rho$ and $t$, but $I_{v,\mathrm{DW}}(t/(2\pi))$ can be used for $\rho > 100$ to reduce the relative error of the approximation even further.

# References


[1] Moutafis, C., Komineas, S., Vaz, C. A. F., Bland, J. A. C. & Eames, P. Vortices in ferromagnetic elements with perpendicular anisotropy. *Physical Review B* **74**, 214406 (2006).

[2] Romming, N., Kubetzka, A., Hanneken, C., von Bergmann, K. & Wiesendanger, R. Field-Dependent

Size and Shape of Single Magnetic Skyrmions. *Physical Review Letters* **114**, 177203 (2015).

[3] Boulle, O. *et al.* Room-temperature chiral magnetic skyrmions in ultrathin magnetic nanostructures. *Nature Nanotechnology* **11**, 449–454 (2016).

[4] Rohart, S. & Thiaville, A. Skyrmion confinement in ultrathin film nanostructures in the presence of Dzyaloshinskii-Moriya interaction. *Physical Review B* **88**, 184422 (2013).

[5] Bogdanov, A. & Hubert, A. Thermodynamically stable magnetic vortex states in magnetic crystals. *Journal of Magnetism and Magnetic Materials* **138**, 255–269 (1994).

[6] Tu, Y.-O. Determination of Magnetization of Micromagnetic Wall in Bubble Domains by Direct Minimization. *Journal of Applied Physics* **42**, 5704–5709 (1971).

[7] Büttner, F., Krüger, B., Eisebitt, S. & Kläui, M. Accurate calculation of the transverse anisotropy of a magnetic domain wall in perpendicularly magnetized multilayers. *Physical Review B* **92**, 054408 (2015).

[8] Winter, J. M. Bloch Wall Excitation. Application to Nuclear Resonance in a Bloch Wall. *Physical Review* **124**, 452–459 (1961).

[9] Cape, J. A. & Lehman, G. W. Magnetic Domain Structures in Thin Uniaxial Plates with Perpendicular Easy Axis. *Journal of Applied Physics* **42**, 5732–5756 (1971).

[10] Lemesh, I., Büttner, F. & Beach, G. S. D. Accurate model of the stripe domain phase of perpendicularly magnetized multilayers. *Physical Review B* **95**, 174423 (2017).

[11] Bogdanov, A. & Hubert, A. The Properties of Isolated Magnetic Vortices. *physica status solidi (b)* **186**, 527–543 (1994).

[12] Ngo, D. T. *et al.* Interfacial tuning of perpendicular magnetic anisotropy and spin magnetic moment in CoFe/Pd multilayers. *Journal of Magnetism and Magnetic Materials* **350**, 42–46 (2014).

[13] Chen, C. W. Fabrication and characterization of thin films with perpendicular magnetic anisotropy for high-density magnetic recording. *Journal of Materials Science* **26**, 3125–3153 (1991).

[14] Woo, S. *et al.* Observation of room-temperature magnetic skyrmions and their current-driven dynamics in ultrathin metallic ferromagnets. *Nature Materials* **15**, 501–506 (2016).

[15] Wu, D. *et al.* Perpendicular magnetic anisotropy and magnetization dynamics in oxidized CoFeAl films. *Scientific Reports* **5**, srep12352 (2015).

[16] Lin, C. & Gorman, G. L. Evaporated CoPt alloy films with strong perpendicular magnetic anisotropy. *Applied Physics Letters* **61**, 1600–1602 (1992).

[17] Emori, S. *et al.* Spin Hall torque magnetometry of Dzyaloshinskii domain walls. *Physical Review B* **90**, 184427 (2014).

[18] Cui, Y. *et al.* Interfacial perpendicular magnetic anisotropy and damping parameter in ultra thin Co2feal films. *Applied Physics Letters* **102**, 162403 (2013).

[19] Mizukami, S. *et al.* Gilbert Damping in Ni/Co Multilayer Films Exhibiting Large Perpendicular Anisotropy. *Applied Physics Express* **4**, 013005 (2011).

[20] Kozioł-Rachwał, A. *et al.* Enhancement of perpendicular magnetic anisotropy and its electric field-induced change through interface engineering in Cr/Fe/MgO. *Scientific Reports* **7**, 5993 (2017).

[21] Moreau-Luchaire, C. *et al.* Additive interfacial chiral interaction in multilayers for stabilization of small individual skyrmions at room temperature. *Nature Nanotechnology* **11**, 444–448 (2016).

[22] An, H. *et al.* Highly (001) oriented L10-CoPt/TiN multilayer films on glass substrates with perpendicular magnetic anisotropy. *Journal of Vacuum Science & Technology A: Vacuum, Surfaces, and Films* **33**, 021512 (2015).

[23] Büttner, F. *et al.* Field-free deterministic ultrafast creation of magnetic skyrmions by spin–orbit torques. *Nature Nanotechnology* **12**, 1040–1044 (2017).

[24] Pai, C.-F. *et al.* Enhancement of perpendicular magnetic anisotropy and transmission of spin-Hall-effect-induced spin currents by a Hf spacer layer in W/Hf/CoFeB/MgO layer structures. *Applied Physics Letters* **104**, 082407 (2014).

[25] Sinha, J. *et al.* Enhanced interface perpendicular magnetic anisotropy in Ta|CoFeB|MgO using nitrogen doped Ta underlayers. *Applied Physics Letters* **102**, 242405 (2013).

[26] Koo, J. W. *et al.* Large perpendicular magnetic anisotropy at Fe/MgO interface. *Applied Physics Letters* **103**, 192401 (2013).

[27] Liu, T., Zhang, Y., Cai, J. W. & Pan, H. Y. Thermally robust Mo/CoFeB/MgO trilayers with strong perpendicular magnetic anisotropy. *Scientific Reports* **4**, srep05895 (2014).

[28] Carcia, P. F., Meinhaldt, A. D. & Suna, A. Perpendicular magnetic anisotropy in Pd/Co thin film layered structures. *Applied Physics Letters* **47**, 178–180 (1985).

[29] Wang, B., Oomiya, H., Arakawa, A., Hasegawa, T. & Ishio, S. Perpendicular magnetic anisotropy and magnetization of L10 FePt/FeCo bilayer films. *Journal of Applied Physics* **115**, 133908 (2014).

[30] Hansen, P., Klahn, S., Clausen, C., Much, G. & Witter, K. Magnetic and magneto-optical properties of rare-earth transition-metal alloys containing Dy, Ho, Fe, Co. *Journal of Applied Physics* **69**, 3194–3207 (1991).

[31] Hansen, P., Clausen, C., Much, G., Rosenkranz, M. & Witter, K. Magnetic and magneto-optical properties of rare-earth transition-metal alloys containing Gd, Tb, Fe, Co. *Journal of Applied Physics* **66**, 756–767 (1989).

[32] Ding, M. & Poon, S. J. Tunable perpendicular magnetic anisotropy in GdFeCo amorphous films. *Journal of Magnetism and Magnetic Materials* **339**, 51–55 (2013).

[33] Brunsch, A. & Schneider, J. Perpendicular magnetic anisotropy in evaporated amorphous GdCo films. *Journal of Applied Physics* **48**, 2641–2643 (1977).

[34] Finley, J. & Liu, L. Spin-Orbit-Torque Efficiency in Compensated Ferrimagnetic Cobalt-Terbium Alloys. *Physical Review Applied* **6**, 054001 (2016).

[35] Lee, C. M. *et al.* Ultrathin (Gd, Tb)-FeCo Films With Perpendicular Magnetic Anisotropy. *IEEE Transactions on Magnetics* **45**, 3808–3811 (2009).

[36] Wu, F. *et al.* Epitaxial Mn2.5ga thin films with giant perpendicular magnetic anisotropy for spintronic devices. *Applied Physics Letters* **94**, 122503 (2009).

[37] Brataas, A. & Hals, K. M. D. Spin-orbit torques in action. *Nature Nanotechnology* **9**, 86–88 (2014).

[38] Li, Z. & Zhang, S. Domain-Wall Dynamics and Spin-Wave Excitations with Spin-Transfer Torques. *Physical Review Letters* **92**, 207203 (2004).

[39] Slonczewski, J. Current-driven excitation of magnetic multilayers. *Journal of Magnetism and Magnetic Materials* **159**, L1–L7 (1996).

[40] Berger, L. Emission of spin waves by a magnetic multilayer traversed by a current. *Physical Review B* **54**, 9353–9358 (1996).

[41] Hayashi, M., Kim, J., Yamanouchi, M. & Ohno, H. Quantitative characterization of the spin-orbit torque using harmonic Hall voltage measurements. *Physical Review B* **89**, 144425 (2014).

[42] Haney, P. M., Lee, H.-W., Lee, K.-J., Manchon, A. & Stiles, M. D. Current induced torques and interfacial spin-orbit coupling: Semiclassical modeling. *Physical Review B* **87**, 174411 (2013).

[43] Krüger, B. *Current-Driven Magnetization Dynamics : Analytical Modeling and Numerical Simulation*. Dissertation, University of Hamburg, Hamburg (2012).

[44] Everschor, K. *et al.* Rotating skyrmion lattices by spin torques and field or temperature gradients. *Physical Review B* **86**, 054432 (2012).

[45] Thiele, A. A. Steady-State Motion of Magnetic Domains. *Physical Review Letters* **30**, 230–233 (1973).

[46] Tomasello, R. *et al.* A strategy for the design of skyrmion racetrack memories. *Scientific Reports* **4**, 6784 (2014).

[47] Jiang, W. *et al.* Direct observation of the skyrmion Hall effect. *Nature Physics* **13**, 162–169 (2016).

[48] Litzius, K. *et al.* Skyrmion Hall effect revealed by direct time-resolved X-ray microscopy. *Nature Physics* **13**, 170–175 (2017).

[49] Malozemoff, A. P. & Slonczewski, J. C. *Magnetic Domain Walls in Bubble Materials* (Academic Press, New York, 1979).

[50] Bogdanov, A. N. & Yablonskii, D. A. Thermodynamically stable "vortices" in magnetically ordered crystals. The mixed state of magnets. *Sov. Phys. JETP* **68**, 101 (1989).

[51] Rößler, U. K., Bogdanov, A. N. & Pfleiderer, C. Spontaneous skyrmion ground states in magnetic metals. *Nature* **442**, 797–801 (2006).

[52] Byrd, P. F. & Friedman, M. D. *Handbook of Elliptic Integrals for Engineers and Physicists* (Springer Berlin Heidelberg, Berlin, Heidelberg, 1954).
